# Stable brain PET metabolic networks using a multiple sampling scheme

**DOI:** 10.1162/NETN.a.23

**Published:** 2025-09-19

**Authors:** Guilherme Schu, Christian Limberger, Wagner S. Brum, Marco Antônio De Bastiani, Yuri Elias Rodrigues, Julio Cesar de Azeredo, Tharick A. Pascoal, Andrea L. Benedet, Sulantha Mathotaarachchi, Pedro Rosa-Neto, Jorge Almeida, Daniele de Paula Faria, Fábio Luiz de Souza Duran, Carlos Alberto Buchpiguel, Artur Martins Coutinho, Geraldo F. Busatto, Eduardo R. Zimmer, for the Alzheimer’s Disease Neuroimaging Initiative

**Affiliations:** Graduate Program in Biological Sciences: Biochemistry, Universidade Federal do Rio Grande do Sul (UFRGS), Porto Alegre, Brazil; Graduate Program in Biological Sciences: Pharmacology and Therapeutics, UFRGS, Porto Alegre, Brazil; Strathclyde Institute of Pharmacy and Biomedical Sciences, University of Strathclyde, Glasgow, UK; Department of Psychiatry and Department of Neurology, University of Pittsburgh, Pittsburgh, PA, USA; Department of Psychiatry and Neurochemistry, The Sahlgrenska Academy at the University of Gothenburg, Mölndal, Sweden; Translational Neuroimaging Laboratory, The McGill University Research Centre for Studies in Aging, Douglas Mental Health Institute, Montreal, QC, Canada; Department of Neurology and Neurosurgery, McGill University, Montreal, QC, Canada; Peter O’Donnell Jr. Brain Institute (OBI), University of Texas Southwestern Medical Centre (UTSW), Dallas, TX, USA; Proaction Laboratory, Faculty of Psychology and Education Sciences, University of Coimbra, Coimbra, Portugal; CINEICC, Faculty of Psychology and Education Sciences, University of Coimbra, Coimbra, Portugal; Laboratory of Nuclear Medicine (LIM 43), Department of Radiology and Oncology, Faculdade de Medicina FMUSP, Universidade de São Paulo, São Paulo, Brazil; Laboratory of Psychiatric Neuroimaging (LIM 21), Department of Psychiatry, Faculdade de Medicina FMUSP, Universidade de São Paulo, São Paulo, Brazil; Nuclear Medicine Service, Centro de Diagnosticos por Imagem, Hospital Sirio-Libanes, São Paulo, Brazil; Department of Pharmacology, Universidade Federal do Rio Grande do Sul (UFRGS), Porto Alegre, Brazil; Brain Institute of Rio Grande do Sul, PUCRS, Porto Alegre, Brazil

**Keywords:** Metabolic brain network, Random sampling, Network stability, Neuroimaging, Positron emission tomography

## Abstract

Interregional communication within the human brain is essential for maintaining functional integrity. A promising approach for investigating how brain regions communicate relies on the assumption that the brain operates as a complex network. In this context, positron emission tomography (PET) images have been suggested as a valuable source for understanding brain networks. However, such networks are typically assembled through direct computation without accounting for outliers, impacting the reliability of group representative networks. In this study, we used brain [^18^F]fluoro-2-deoxyglucose PET data from 1,227 individuals in the Alzheimer’s disease (AD) continuum from the Alzheimer’s Disease Neuroimaging Initiative cohort to develop a novel method for constructing stable metabolic brain networks that are resilient to spurious data points. Our multiple sampling scheme generates brain networks with greater stability compared with conventional approaches. The proposed method is robust to imbalanced datasets and requires 50% fewer subjects to achieve stability than the conventional method. We further validated the approach in an independent AD cohort (*n* = 114) from São Paulo, Brazil (Faculdade de Medicina da Universidade de São Paulo). This innovative method is flexible and improves the robustness of metabolic brain network analyses, supporting better insights into brain connectivity and resilience to data variability across multiple radiotracers for both health and disease.

## INTRODUCTION

Brain cells rely on glucose as a primary source of energy, making glucose metabolism crucial for neurotransmission and synaptic function (Bélanger et al., 2011). [^18^F]fluoro-2-deoxyglucose positron emission tomography ([^18^F]FDG-PET) has been suggested as a measure of regional glucose consumption, linked to the local intensity of brain glutamatergic and astrocyte activity. This assumption serves as the basis for the interpretation of [^18^F]FDG-PET imaging (Chételat et al., 2020; Rocha et al., 2022; Zimmer et al., 2017). Since the 1970s, cerebral glucose metabolism—indexed by semiquantitative (standardized uptake value [SUV] or standardized uptake value ratio [SUVR]) or quantitative measurements—has been used to estimate in vivo regional tissue glucose metabolism in normal and pathological states (Bohnen et al., 2012; Reivich et al., 1979). More recently, derivation of metabolic brain networks (MBNs) from intersubject [^18^F]FDG-PET data has been proposed as a novel manner to gain additional information about the energetic architecture of the brain (Bullmore & Sporns, 2009, 2012; Friston, 2011).

MBNs have been firstly introduced by Horwitz et al. (1984) and are typically represented by weighted graphs whose nodes are associated with predefined brain regions in which the edges reveal the network coupling (Bazinet et al., 2023). Currently, MBNs usually rely on the computation of linear correlations (i.e., Pearson correlation coefficients) of [^18^F]FDG-PET measures of brain regions across subjects. This straightforward methodology has been used to identify metabolic architectural changes in Alzheimer’s disease (AD; Arnemann et al., 2017; Grady et al., 2001; Horwitz et al., 1987; Yao et al., 2015), Parkinson’s disease (Ge et al., 2014; C. Huang et al., 2007), cognitive impairment (Carbonell et al., 2014; Chen et al., 2018), diabetes mellitus (Qiu et al., 2016), aging (Horwitz et al., 1986; Liu et al., 2014), and long COVID (Martini et al., 2025).

Although MBNs constructed from intersubject correlations continues to be explored, evaluations of the variability of such networks remain little explored, leading to substantial inconsistencies evidenced across similar [^18^F]FDG-PET studies and other neuroimaging modalities (Di et al., 2017; Yao et al., 2018). We hypothesize that intersubjects linear group correlations are highly unstable—susceptible to outliers, sample size, and data imbalance—and may be hampering reliable and reproductible construction of MBNs. Here, we present an innovative general methodology for constructing more stable brain networks with PET imaging data, based on a multiple sampling (MS) scheme, in association with a more conservative method for multiple comparisons correction.

## MATERIALS AND METHODS

### Data Source

Data used in the preparation of the present study were obtained from the Alzheimer’s Disease Neuroimaging Initiative (ADNI) database (https://adni.loni.usc.edu). The ADNI was launched in 2003 as a public-private partnership, led by Principal Investigator Michael W. Weiner, MD. The primary goal of ADNI has been to test whether serial magnetic resonance imaging (MRI), positron emission tomography (PET), other biological markers, and clinical and neuropsychological assessment can be combined to measure the progression of mild cognitive impairment (MCI) and early Alzheimer’s disease (AD). For up-to-date information, see www.adni-info.org.

The ADNI’s research population consists of cognitively unimpaired (CU), early or late mild cognitive impairment (MCI), and Alzheimer’s clinical syndrome (AD) individuals. The follow-up duration of each group is specified in the protocols for ADNI-1, ADNI-2, and ADNI-GO study phases. Furthermore, we used a second cohort from the Faculdade de Medicina da Universidade de São Paulo (FMUSP) to validate our sampling strategy (Coutinho et al., 2020).

The institutional review boards of FMUSP and all sites participating in the ADNI provided review and approval of the data collection protocols. Written informed consent was obtained from all participants at each site.

### Study Participants Selection

We selected 1,227 individuals based on their clinical diagnosis (352 CU, 641 MCI, and 234 AD) who underwent [^18^F]FDG-PET and structural MRI scans in ADNI-1, ADNI-GO, and ADNI-2 phases of the ADNI project, and 114 individuals (35 CU, 42 MCI, and 37 AD) from the FMUSP cohort. Demographic characteristics of the groups are presented in Table 1. Additionally, to tune the proposed method parameters, the ADNI dataset was split into training and test sets as depicted in Table 2. The training set used 300 MCI individuals from ADNI to identify the number of bootstrap samples to obtain a representative matrix. The test set was composed of data from ADNI (352 CU, 341 MCI, and 234 AD) and FMUSP (35 CU, 42 MCI, and 37 AD).

**Table 1.** Cohorts demographics

**ADNI cohort**				
	**CU**	**MCI**	**AD**	***p* value**
Number of subjects (%)	352 (28.7%)	641 (52.2%)	234 (19.1%)	
Age, y, mean (*SD*)	74.3 (5.6)	72.7 (7.5)	74.3 (7.7)	CU-MCI = 0.289
				MCI-AD = 0.452
				CU-AD = 0.997
Male, no (%)	177 (50.3)	380 (59.3)	135 (57.7)	**CU-MCI = 0.015**
				MCI-AD = 0.631
				CU-AD = 0.093
Education, y, mean (*SD*)	16.3 (2.8)	16 (2.8)	15.5 (2.9)	CU-MCI = 0.912
				MCI-AD = 0.680
				CU-AD = 0.489
MMSE, mean (*SD*)	29 (1.2)	27.8 (1.7)	23.7 (2.4)	**CU-MCI < 0.0001**
				**MCI-AD < 0.0001**
				**CU-AD < 0.0001**
**FMUSP cohort**				
	**CU**	**MCI**	**AD**	***p* value**
Number of subjects (%)	35 (30.7%)	42 (36.8%)	37 (32.5%)	
Age, y, mean (*SD*)	70.9 (5.7)	73 (6)	73.9 (7.3)	CU-MCI = 0.124
				MCI-AD = 0.502
				**CU-AD = 0.048**
Male, no (%)	10 (0.2)	10 (0.3)	13 (0.4)	CU-MCI = 0.620
				MCI-AD = 0.311
				CU-AD = 0.285
Education, y, mean (*SD*)	10.8 (5.4)	10.1 (4.8)	9.5 (5)	CU-MCI = 0.703
				MCI-AD = 0.521
				CU-AD = 0.311
MMSE, mean (*SD*)	28 (1.7)	26.5* (2.2)	21.7* (4)	**CU-MCI = 0.003**
				**MCI-AD < 0.0001**
				**CU-AD < 0.0001**

* Missing value of one subject. Group demographic comparisons were performed with chi-square test for categorical variables and Dunn’s test if significant after the Kruskal–Wallis test for continuous variables.

**Table 2.** ADNI cohort dataset organization

	**No. individuals × no. VOIs**	**Group**
**Training set**	300 × 72	MCI
	352 × 72	CU
**Test set**	341 × 72	MCI
	234 × 72	AD

### Neuroimaging Methods

MRI and PET acquisitions followed the ADNI’s protocols (https://adni.loni.usc.edu/help-faqs/adni-documentation/). MRI T1-weighted images were preprocessed for gradient unwarping and intensity normalization (Eskildsen et al., 2012). The T1-weighted images were then processed using the CIVET image-processing pipeline and registered using a nine-parameter affine transformation and nonlinearly spatially normalized to the Montreal Neurological Institute’s 152 brain template (MNI 152) template (Zijdenbos et al., 2002). [^18^F]FDG-PET images were preprocessed to have an effective point spread function of full width at half maximum of 8 mm. Subsequently, linear registration and nonlinear normalization to the MNI 152 template were performed with the linear and nonlinear transformation derived from the automatic PET to MRI transformation and the individual’s anatomical MRI coregistration. [^18^F]FDG-PET SUVR maps were generated using pons as the reference region (Pascoal, Mathotaarachchi, Mohades et al., 2017). Further details on the processing pipeline used on ADNI and FMUSP cohorts can be found elsewhere (Coutinho et al., 2020; Pascoal, Mathotaarachchi, Shin, et al., 2017).

### Construction of MBNs Using an MS Scheme

Let *X* ∈ *ℝ*^*N*×*d*^ be a *N* × *d* dataset matrix containing PET SUVR values of *N* subjects for *d* volumes of interest (VOIs) of a given group. The proposed MS scheme works by generating *n* samples *Y*^1^, …, *Y^n^* of the dataset *X*. Each bootstrap sample *Y^k^* ∈ *ℝ*^*N*×*d*^ is a dataset matrix that contains duplicate rows of the original dataset (i.e., *Y^k^* ⊆ *X*). In this context, each column of *Y^k^* is denoted by the vector $y_{j}^{k}$ (with 1 ≤ *j* ≤ *d*).

Given the aforementioned notations, we can construct the adjacency matrix *M^k^* ∈ *ℝ*^*d*×*d*^, associated with the dataset *Y^k^*, by computing the Pearson linear correlation coefficients between all columns of matrix *Y^k^*, with *p, q* = 1, …, *d*, as follows:

$$r_{p,q}^{k}=\frac{\sum_{i=1}^{N}\left(y_{i,p}^{k}-\bar{y_{p}^{k}}\;\right)\left(y_{i,q}^{k}-\bar{y_{q}^{k}}\;\right)}{\sqrt{\sum_{i=1}^{N}{\left(y_{i,p}^{k}-\bar{y_{p}^{k}}\;\right)}^{2}}\sqrt{\sum_{i=1}^{N}{\left(y_{i,q}^{k}-\bar{y_{q}^{k}}\;\right)}^{2}}}$$ (1)

where $y_{i,p}^{k}$ and $y_{i,q}^{k}$ correspond to the *i*-th element of the vectors $y_{p}^{k}$ and $y_{q}^{k}$, $\bar{y_{p}^{k}}=\frac{1}{N}\sum_{i=1}^{N}y_{i,p}^{k}$ and $\bar{y_{q}^{k}}=\frac{1}{N}\sum_{i=1}^{N}y_{i,q}^{k}$ are the mean values of the column vectors $y_{p}^{k}$ and $y_{q}^{k}$, respectively.

For each dataset *Y^k^* generated by the MS scheme, we compute, using Equation 1, a weighted undirected network $M^{k}={\left[r_{p,q}^{k}\right]}_{d\times d}$. Given a collection of generated networks Φ = {*M*^1^, *M*^2^, …, *M^n^*} for a group of interest, we then decide which of these networks can be chosen as the representative network of the group. A direct approach is to compute the mean matrix $\bar{M}$ (i.e., $\frac{1}{n}\sum_{k=1}^{n}M^{k}$) and identify which of the matrices in the set Φ best approximates $\bar{M}$ according to some metric. Hence, we define a representative network M of the group as:

$$M=\underset{1\leq k\leq n}{\text{argmin}}\left\{{\left\|M^{k}-\bar{M}\right\|}_{F}\right\},$$ (2)

where ‖ . ‖*_F_* is the Frobenius norm. With this formulation, we seek to find the adjacency matrix M that best approximates a generated matrix *M^k^* from the mean matrix $\bar{M}$.

**Figure 1.**
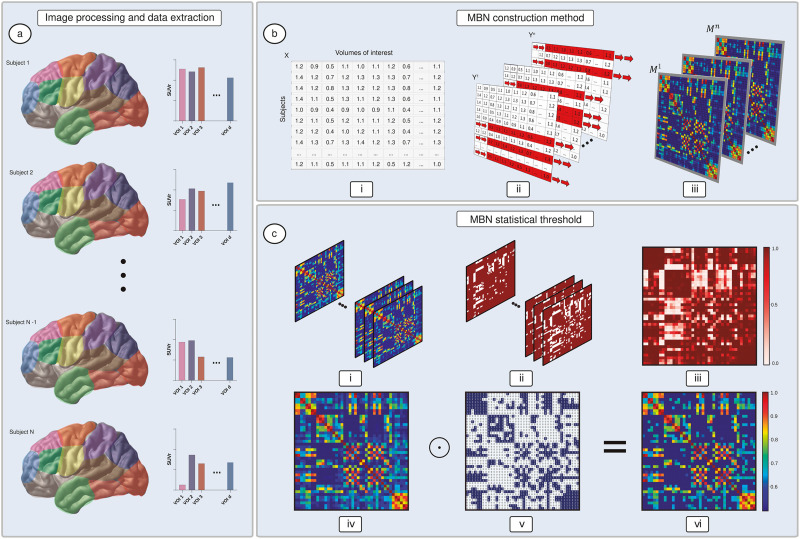
Illustration of the MS scheme. [^18^F]FDG-PET SUVR are computed for *d* VOIs and *N* subjects—as shown in (A)—generating a dataset matrix for a group of interest. The original dataset matrix *X* ∈ *ℝ*^*N*×*d*^ shown in (B.i) is bootstrap sampled *n* times, generating the datasets *Y*^1^, …, *Y^n^* shown in (B.ii). For each of the sampled datasets *Y*^1^, …, *Y^n^*, adjacency matrices *M*^1^, …, *M^n^* are constructed by computing intersubject Pearson correlation coefficients (B.iii). The collection of FDR corrected adjacency matrices *M*^1^, …, *M^n^* shown in (C.i) have their entries evaluated using Equation 3, generating the binary matrices shown in (C.ii). An average degree probability distribution matrix $\dot{P}$ (i.e., a Pmap), shown in (C.iii), is computed by averaging the collection of binary matrices. Given a defined threshold *θ*, matrix is then thresholded using Equation 4, generating a threshold matrix $\dot{T}$ shown in (C.v). Finally, the FDR corrected representative matrix M shown in (C.iv) is thresholded by computing the Hadamard product between M and $\dot{T}$, which results in the corrected matrix shown in (C.vi).

Algorithm 1 can be used iteratively to construct group representative networks. In practice, a sample *Y^k^* of the original dataset *X* can be generated using different sampling approaches, such as bootstrap and subsampling methods. Likewise, different criteria than the one presented in Equation 2 (i.e., the mean matrix) can be used to choose the representative network of the group of interest. Additionally to the mean criterion (see Equation 2), in MS Scheme Does Not Depend on the MBN Construction Criteria section, we test the median criterion and a generalization of the Bayesian mode criterion proposed in our previous work (Schu & Scharcanski, 2018). Figure 1 summarizes the MS approach. Finally, given the collection of generated networks Φ for a group of interest, the representative MBN is chosen and corrected for multiple comparisons using false discovery rate (FDR; Benjamini & Hochberg, 1995). For more information about how to optimally set a subsampling scheme or mode criterion, see the Supporting Information.

**Algorithm 1.** MBN construction. 1: **procedure** build_network(*X*, *n*).

2: **for** *k* ← **to** *n* **do**

3:  *Y*^*k*^ ← randomly bootstrap sample from *X*.

4:  **for** *p* ← 1 **to** *d* **do**

5:  **for** *q* ← 1 **to** *d* **do**

6:   Compute *M*^*k*^ using Equation 1.

7: Compute M using Equation 2 and correct it with FDR.

8: **return** M

### Network Threshold Using Probability Maps

Here, we propose a novel approach for thresholding the group representative MBNs in addition to the FDR method. We generate a probability map (Pmap) based on the average degree distribution computed from *n* bootstrap samples. We verify which edges are more likely to occur after sampling the data randomly multiple times. With this scheme, if the probability of an edge to occur is higher than a threshold *θ*, the edge should be retained; otherwise, it should be excluded. Explicitly, for *p, q* = 1, …, *d*, we define the entries of the probability matrix $\dot{P}$ as:

$${\dot{P}}_{p,q}=\frac{1}{n}\sum_{k=1}^{n}\hat{B}\left(r_{p,q}^{k}\neq 0\right),$$ (3)

where $r_{p,q}^{k}$ corresponds to elements of *M^k^* (corrected by FDR) and the function $\hat{B}\left(.\right)$ evaluated for an arbitrary logical statement *z* given by:

$$\hat{B}\left(z\right)=\left\{\begin{array}{c} 1,\;\text{if}\;z\;\text{is}\;\text{True},\\ 0,\;\text{if}\;z\;\text{is}\;\text{False}. \end{array}\right.$$ (4)

Given a choice for *θ*, we generate a threshold matrix using the computed Pmap as:

$${\dot{T}}_{p,q}=\hat{B}\left({\dot{P}}_{p,q}>\theta \right).$$ (5)

Finally, we threshold the FDR corrected representative matrix M by computing the Hadamard product with the threshold matrix $\dot{T}$ (i.e., M ⊙ $\dot{T}$). In practice, we define *θ* = 1 − *α*, where *α* is the statistical threshold typically used in multiple comparison methods such as FDR. Figure 1C shows a representation of the proposed threshold scheme. Using the collection of networks generated with the MS approach (Figure 1C.i), we test whether their entries are different from zero, generating the binary matrices showed in Figure 1C.ii. Computing the sum over all binary matrices and dividing their elements by *n*, we generate the probability matrix $\dot{P}$ (Figure 1C.iii). Finally, the representative matrix M corrected by FDR (Figure 1C.iv) is thresholded by computing the Hadamard product between Mand $\dot{T}$ (Figure 1C.v), which results are in the corrected matrix (Figure 1C.vi).

### Network Model Outlier Attack

Here, we adapted the node attack strategy used in Albert et al. (2000) and proposed an attack method that is driven by the inclusion of outliers to a group dataset aiming to evaluate MBNs stability. More specifically, we generate *Q* perturbed versions ${\tilde{M}}_{1},\ldots ,M_{Q}$ of the original representative matrix M. In this model, for each perturbed network, we introduce *L* outliers to the dataset *Y^q^* (that has producedM) to generate a perturbed network $\tilde{M}$. We defined outliers as data points randomly sampled from datasets belonging to the groups being compared, which are introduced in the dataset of a group of interest. In this model, if one would make an attack to the CU group, for instance, it would consist of randomly sampling data points from the MCI and AD groups to later introduce it into the CU dataset. In our experiments, the attacks were programmed in a way that given the parameter percentage of outliers (*P*_*o*_), half of the outliers introduced to the dataset of interest (e.g., CU) are from one group (e.g., MCI), and half are from the other group (e.g., AD). We consider this a valid attack model because the metabolic patterns across different groups generally show subtle differences in absolute value, which may not be easily detected by standard outlier-removal methods. In the experimental setup we have defined *P*_*o*_ ∈ [2%, 5%, 8%] and the total number of attacks equal to 256.

Likewise, it is possible to apply this procedure to generate perturbed intersubject correlation matrices with no random samples (what we refer here as the conventional method). In this case, the same outliers used to build perturbed representative matrices constructed using the MS scheme are included in the original dataset *X* ∈ *ℝ*^*N*×*d*^, generating a perturbed dataset $\tilde{X}\in {\mathbb{R}}^{\left(N+L\right)\times d}$. An intersubject correlation matrix and its perturbed version can then be obtained by computing the Pearson linear correlation coefficients using *X* and $\tilde{X}$ followed by FDR correction.

### Network Stability Measures

We address the problem of measuring the stability of a MBN by evaluating the similarity between an arbitrary group representative network M and its perturbed version $\tilde{M}$. We argue that alterations in the topology and characteristics of the network, provoked by spurious data samples, are directly associated with the stability of the network. To quantitatively evaluate the stability of a network and its perturbed version, we explore four different metrics: Hausdorff distance (dH), Frobenius distance (dF), Euclidean distance (dE), and Canberra distance (dC).

The Frobenius norm can be computed directly from the network and its perturbed version (i.e., *M* and $\tilde{M}$). This norm is a common measure used to compute matrix similarities, with lower values (i.e., closer to 0) indicating a higher degree of network stability (i.e., matrix similarity). Likewise, we investigate the use of the dH to estimate network stability. Given an adjacency matrix, *M* = [***m***_1_, …, ***m****_d_*], and a perturbed version of it, $\tilde{M}=\left[{\tilde{m}}_{1},\ldots ,{\tilde{m}}_{d}\right]$, the dH between these two matrices is defined as (Huttenlocher et al., 1993):

$$dH\left(M,\tilde{M}\right)=\text{max}\left(h\left(M,\tilde{M}\right),h\left(\tilde{M},M\right)\right),$$ (6)

where the directional dH *h*(.) is defined as:

$$h\left(M,\tilde{M}\right)=\underset{1\leq i\leq d}{\text{max}}\underset{1\leq j\leq d}{\text{min}}{\left\|m_{i}-{\tilde{m}}_{j}\right\|}_{2},$$ (7)

and ‖ . ‖_2_ is the Euclidean norm.

In addition, we also investigate the dE and the dC as measures of network stability. In this scheme, the similarity of adjacency matrices is evaluated in the vector space spanned by the network graph theoretical measures. For each pair of networks *M*, and its perturbed version $\tilde{M}$, we compute feature vectors *f* = [*ge*, *ac*, *ad*, *as*, *d*, *acc*] and $\tilde{f}=\left[\tilde{g}e,\tilde{a}c,\tilde{a}d,\tilde{a}s,\tilde{d},\tilde{a}cc\right]$, respectively. The feature vector entries correspond to graph theoretical measures known as the global efficiency (*ge*), assortativity coefficient (*ac*), average degree (*ad*), average strength (*as*), density (*d*), and average clustering coefficient (*acc*). For a complete description of how to carry out their computation, see further reference (Rubinov & Sporns, 2010). Given the aforementioned definitions, one can evaluate the stability of a network in terms of feature vectors with the Euclidean norm:

$$dE\left(f,\tilde{f}\right)={\left\|f-\tilde{f}\right\|}_{2},$$ (8)

and in terms of the dC:

$$dC\left(f,\tilde{f}\right)=\sum_{i}\frac{\left|f_{i}-{\tilde{f}}_{i}\right|}{f_{i}\vee +{\tilde{f}}_{i}\vee ,}$$ (9)

where | . | denotes the *L*_1_ norm and, *f*_*i*_ and ${\tilde{f}}_{i}$ correspond to the *i*-th elements of vectors *f* and $\tilde{f}$, respectively. The lower the values (i.e., closer to 0) for *dE* and *dC* the higher the similarity between features, indicating a higher degree of network stability (Soundarajan et al., 2014).

### Data Imbalance and Number of Subjects

Group differences in the sample size (i.e., imbalance distribution of data) are common when studying the AD spectrum. Not rarely, the number of subjects diagnosed as belonging to the MCI group is greater than the number of subjects belonging to the CU or AD groups in the publicly available databases.

As described in the Neuroimaging Methods section, after constructing a representative MBN for a group of interest with the MS scheme, we correct the adjacency matrix with the FDR and the Pmap methods, which depend on the threshold *α*. In data analysis, the *p* value tends to decrease when the sample size increases (Lin et al., 2013). Thus, we also investigate the effects of data imbalance in the construction of MBNs by randomly undersampling and oversampling the [^18^F]FDG-PET data in our experiments. The random undersampling technique consists in removing instances from the larger groups until achieving the same size between all groups (Rodrigues et al., 2017). On the contrast, oversampling techniques typically generate synthetic patterns based on the vicinity of the existing data points, being carried until the smaller groups approximate the largest group’s size. In this work, we have used the adaptive synthetic sampling approach for imbalanced learning (ADASYN; He et al., 2008) method in our experiments.

Likewise, the construction of more reproducible MBNs tends to be very dependent on the total number of subjects in a dataset. A great number of subjects produce better estimates of the underlying distribution of the data, which in turn can be used to construct more reliable and reproducible MBNs. In our experiments, we also evaluate the effects of the number of subjects (*ns*) as a function of the stability of the MBNs. For these experiments, we perturbed the MBNs 256 times, set *P*_*o*_ ∈ [2%, 5%, 8%] and let *ns* ∈ [10, 15, 20, …, 200].

### Parameter Tuning

As previously described in the Construction of MBNs 171 Using an MS Scheme section, the proposed MS bootstrap construction scheme requires as input the number of samples (*n*), which defines the number of different networks that will be constructed to estimate later the representative matrix of a given group of interest. Aiming at optimizing our method, we assumed that the parameter *n* was independent of any group, which entails those optimizations carried out in the space of parameters of one group (e.g., MCI) could be applied to the other groups (e.g., CU and AD) with no great losses in performance. The adopted optimization scheme iteratively computed the Bhattacharyya distance (dB) between the degree distribution (i.e., the normalized histogram of edges connecting paired nodes) of the networks generated with the MS bootstrap scheme when *n* =*k*, against the degree distribution of MBNs generated when *n* =*k* + 100 (with *k* ∈ [100, 200, …, 9900]). We optimized our setup by choosing the parameter *n* that minimized the computed dB values.

## RESULTS

### Asymptotic Decay of MBN Degree Distribution Variability

**Figure 2.**
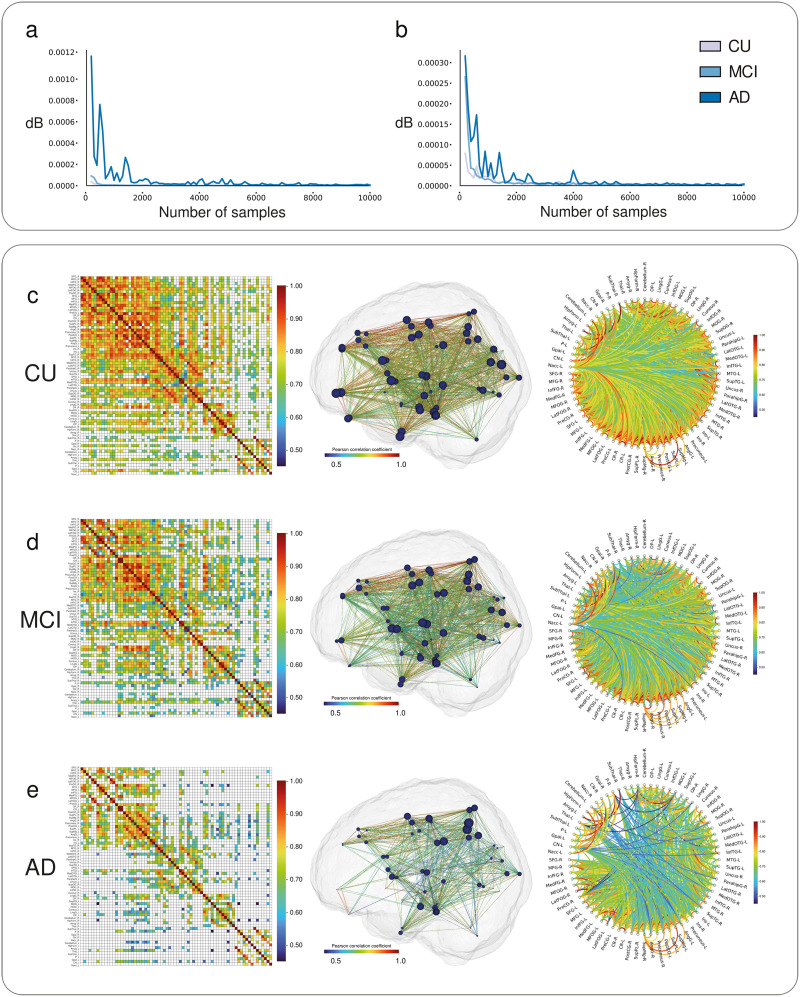
MS bootstrap scheme assembled metabolic brain networks with optimal parameters setup. To define the optimal number of samples *n*, dB values were computed between distributions generated with the MS bootstrap scheme when *n* = *k*, against distributions computed when *n* = *k* + 100 (with *k* ∈ [100, 200, …, 9900]). dB distribution values for CU, MCI, and AD mean representative MBNs degree distributions (A) and weights distributions (B) are displayed as a function of the number of samples. Adjacency matrices of correlation coefficients between brain regions, 3D brain surfaces displaying MBNs architecture and circle plot visualizations are shown for CU (C), MCI (D), and AD (E) groups.

Using the training set, our experiments have revealed that the dB values are minimized when *n* = 9,300. Using the test set, we also investigated the similarity of degree distributions and weights distribution (i.e., the normalized histogram of weights connecting paired nodes divided into 100 bins) for all groups. As *n* → ∞, the variability in both the degree distribution and the weight distribution asymptotically approaches zero for all groups (Figure 2A–B). This decay pattern was observed for the multiple random subsampling scheme as well (see Supporting Information Figure S1). In addition, the AD group requires larger *n* values to present dB measures comparable with CU and MCI groups when analyzing MBNs degree distribution. Moreover, we noted that the optimized parameter *n* obtained for the training set, also produced consistently low dB values (i.e., close to zero) for the CU, MCI, and AD groups in the test set. Figure 2C–E shows the stable MBNs constructed with optimized MS scheme for CU, MCI, and AD groups, respectively. To evaluate the impact of covariates, MBNs have been corrected for age and sex (Supporting Information Figure S2). The networks remained strikingly similar, supporting their stability as group representatives. A list with all VOIs used to construct the MBNs can be found in Supporting Information Table S1.

### Pmap Threshold Excludes MBNs Weakly Linked Edges

**Figure 3.**
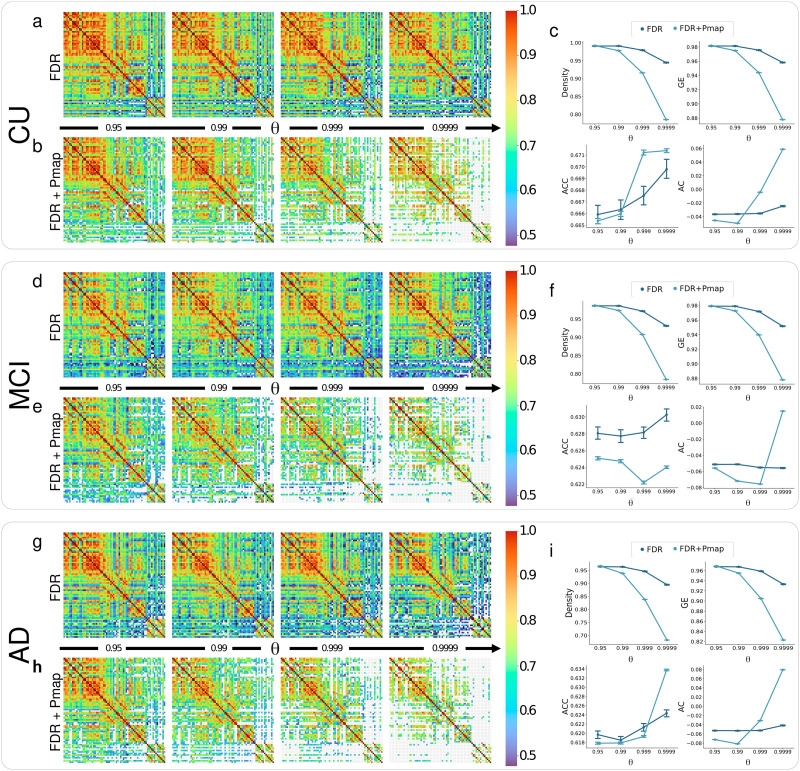
Impact of the proposed thresholding method on mean representative MBNs for CU, MCI, and AD groups. Panels (A) and (B) show, respectively, the mean MBN for the CU group corrected by FDR and by FDR + Pmap at significance levels *α* = 0.05, 0.01, 0.001, and 0.0001. Panels (D) and (E) display the mean MBN for the MCI group with FDR and FDR + Pmap corrections, while panels (G) and (H) show the mean MBN for the AD group with the same corrections. Panels (C), (F), and (I) illustrate the behavior of graph measures—density, *ge*, *ac*, and *acc*—as a function of significance levels for the CU, MCI, and AD groups.

The Pmap threshold (FDR + Pmap**)** revealed to be more conservative (i.e., it tends to eliminate more edges connecting pairs of nodes) when compared with the FDR method alone. As *α* decreases, the proposed threshold tends to maintain only edges strongly linked—edges with weight values closer to |1|—for all groups. We also identified that the MBNs global graph measures are also impacted by different *α* values. More specially, lower *α* values led to decreased *d* and *ge* measures but increased assortativity (*ac*) and *acc*. Variations in the characteristics of the MBNs when comparing FDR + Pmap with FDR alone can be directly observed by inspecting the adjacency matrices of groups CU (Figure 3A–B), MCI (Figure 3D–E), and AD (Figure 3G–H) as a function of the parameter *θ*.

### MS Scheme Does Not Depend on the MBN Construction Criteria

**Figure 4.**
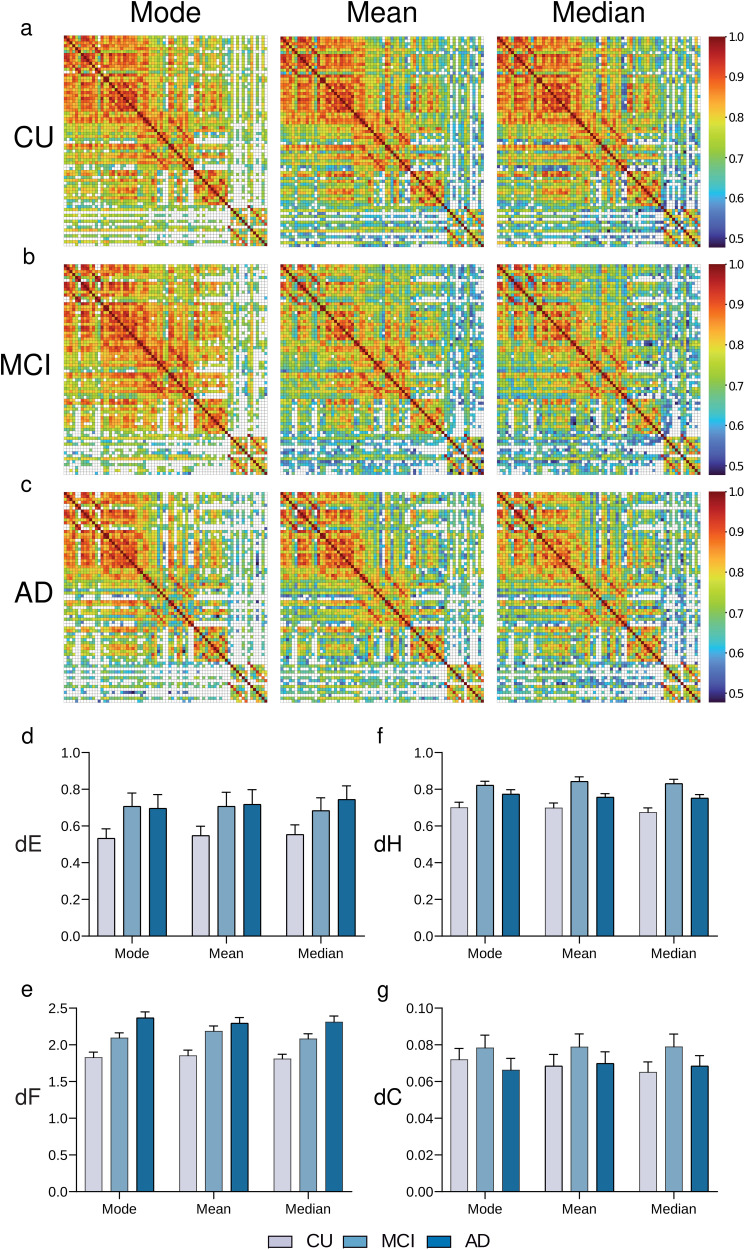
Group MBNs constructed with the mode, mean, and median criteria. Representative matrices using mode, mean, and median as construction criteria for CU (A), MCI (B), and AD (C) groups. The MBNs stability evaluation using the dE, the dF, the dH, and the dC, are shown in (D), (E), (F), and (G), respectively.

In the Neuroimaging Methods section, we have defined the representative MBN for a group of interest as the one that best approximates the mean matrix using the Frobenius norm. We also explored other reasonable choices for the group representative matrix, namely, the mode (maximum density) matrix and the median matrix for groups CU, MCI, and AD (Figure 4A–C). Statistical analysis revealed that the mode, mean, and median criteria do not differ from each other in terms of stability measures dE, dF, dH, and dC (Figure 4D–G). We computed 4 two-way analyses of variance (ANOVAs), one for each stability measure, with a group factor (with three levels: CU, MCI, and AD) and criteria factor (with three levels: mode, mean, and median), followed by Bonferroni correction. No significant alterations were found varying the MBN construction criteria for dE (*F*_(2,2295)_ = 0.1639, *p* = 0.8488), dF (*F*_(2,2295)_ = 1.079, *p* = 0.3403), dH (F_(2,2295)_ = 1.143, *p* = 0.3189), and dC (*F*_(2,2295)_ = 0.2085, *p* = 0.8118). In contrast, the group factor significantly modified the stability measures dE (*F*_(2,2295)_ = 23.09, *p* < 0.0001), dF (*F*_(2,2295)_ = 135.1, *p* < 0.0001), dH (*F*_(2,2295)_ = 101.1, *p* < 0.0001), and dC (*F*_(2,2295)_ = 10.36, *p* < 0.0001; Figure 4D–G).

### Stable MBNs Are Robust to the Data Imbalance Problem

**Figure 5.**
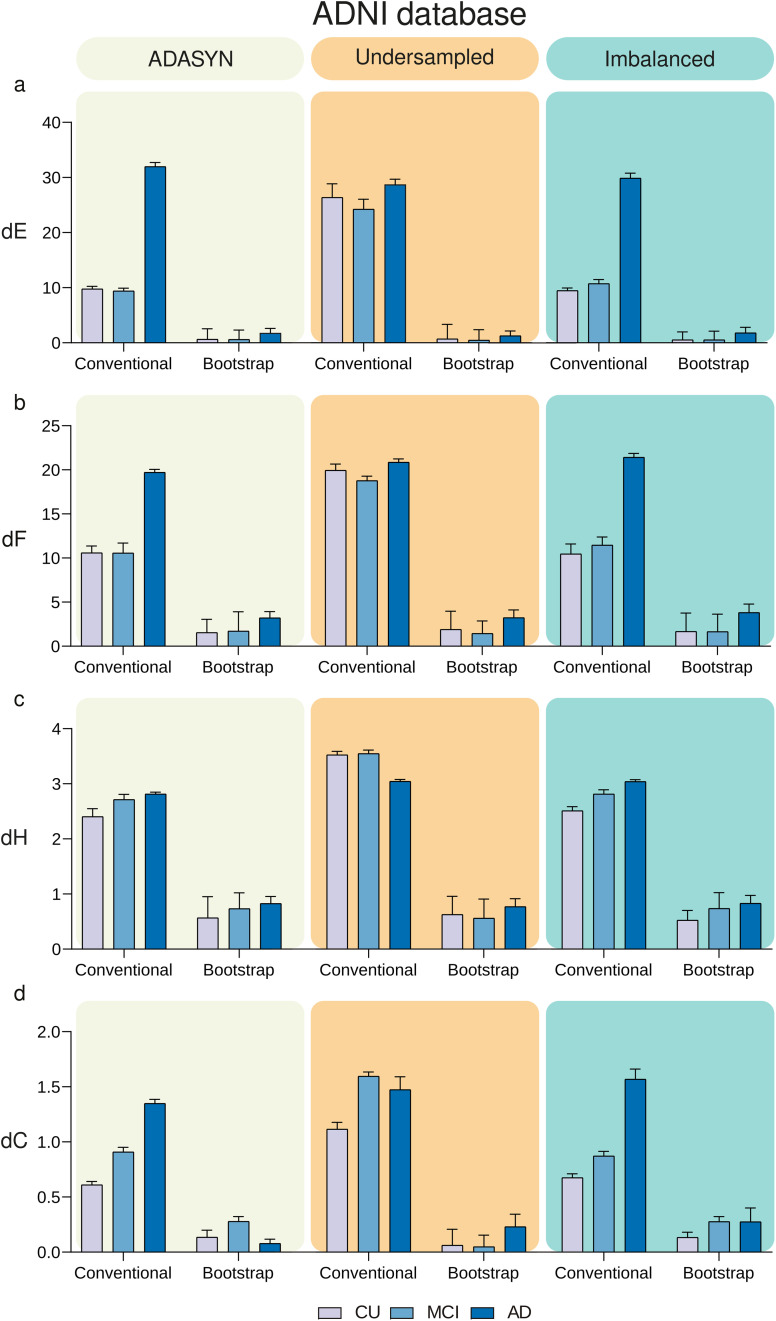
Comparison of MBNs stability between the conventional and the MS bootstrap method across different balance schemes using the ADNI cohort. The CU, MCI, and AD groups underwent 256 network outlier attacks (*P*_*o*_ = 2%) using balance schemes: ADASYN (left column), Undersampled (middle column), and Imbalanced (right column). MS bootstrap MBNs were generated using the mean matrix criterion with *α* = 0.0001, and *n* set to 9,300. The stability of MBNs was evaluated using dE, dF, dH, and dC, shown in panels (A), (B), (C), and (D), respectively, for both conventional and bootstrap methods.

**Figure 6.**
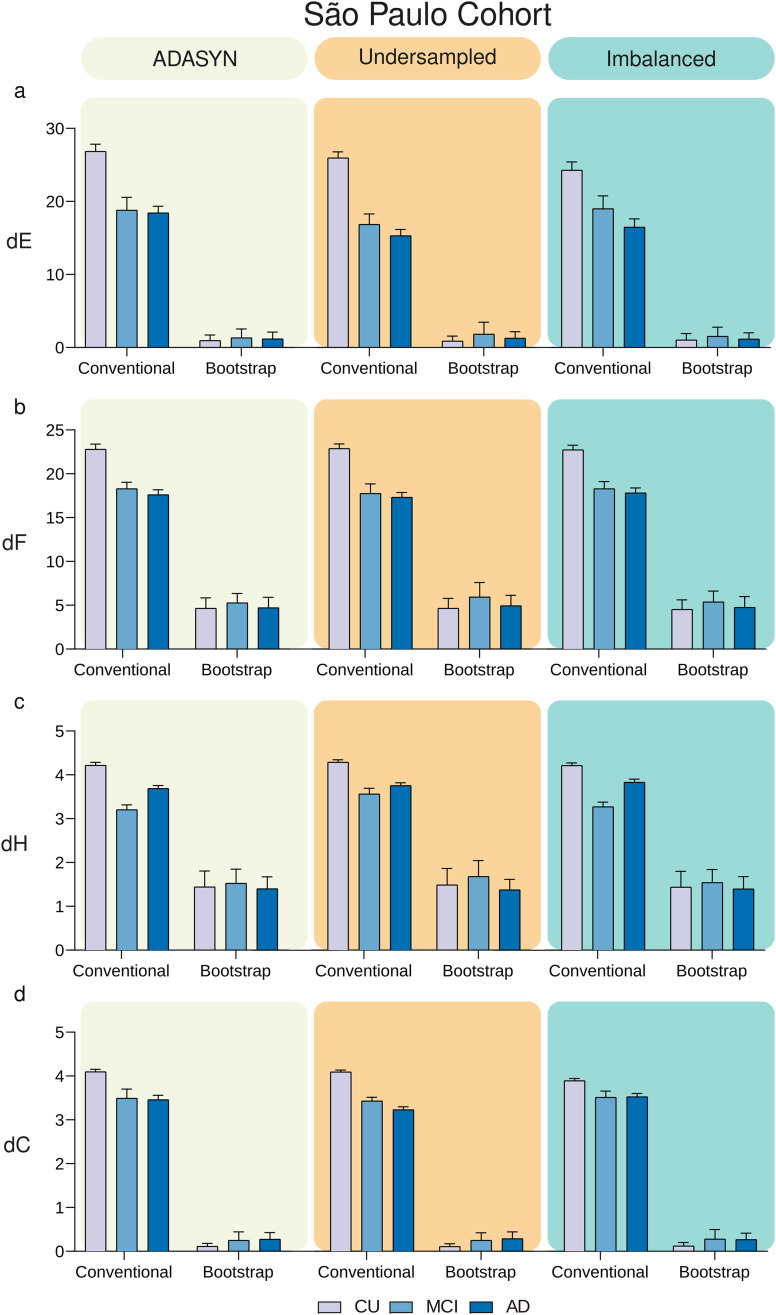
Comparison of MBNs stability between the conventional and the MS bootstrap method across different balance schemes using the FMUSP cohort. The CU, MCI, and AD groups underwent 256 network outlier attacks (*P*_*o*_ = 2%) using balance schemes: ADASYN (left column), Undersampled (middle column), and Imbalanced (right column). MS bootstrap MBNs were generated using the mean matrix criterion with *α* = 0.0001, and *n* set to 9,300. The stability of MBNs was evaluated using dE, dF, dH, and dC, shown in panels (A), (B), (C), and (D), respectively, for both conventional and bootstrap methods.

Network outlier attacks (with percentage of outliers of 2%) using the ADNI cohort revealed that MBNs constructed with the MS bootstrap scheme are more stable than conventional MBNs in different contexts of group imbalance (Figure 5A–D). We performed 12 two-way ANOVAs, one for each stability measure (dE, dF, dH, dC) and one for each data balance setup (ADASYN, Undersampled, Imbalanced), followed by Bonferroni correction. For each ANOVA, the variation sources were the group (with three levels: CU, MCI, and AD) and the MBN construction method (with two levels: conventional and bootstrap). All 12 two-way ANOVAs revealed that the construction method altered the stability measures significantly (*p* < 0.0001). Likewise, group and the interaction group × method were found to alter the stability measures dE, dH, dF, and dC significantly (*p* < 0.0001). Full ANOVA results can be found in Supporting Information Table S2. Likewise, the MS bootstrap scheme revealed to be more stable against network outlier attacks with 5% and 8% of outliers (Supporting Information Figures S3 and S4). Regarding the imbalance issue, the multiple random subsampling scheme was more stable than the conventional method against network outlier attacks with 2%, 5%, and 8% of outliers (Supporting Information Figures S5–S7). Furthermore, the same network outlier attack strategy was used in the FMUSP cohort, and the MBNs generated with the proposed method were also more stable in all metrics (dE, dH, dF, and dC with *p* < 0.0001; Figure 6A–D).

### Construction of Stable MBNs With Small Datasets

**Figure 7.**
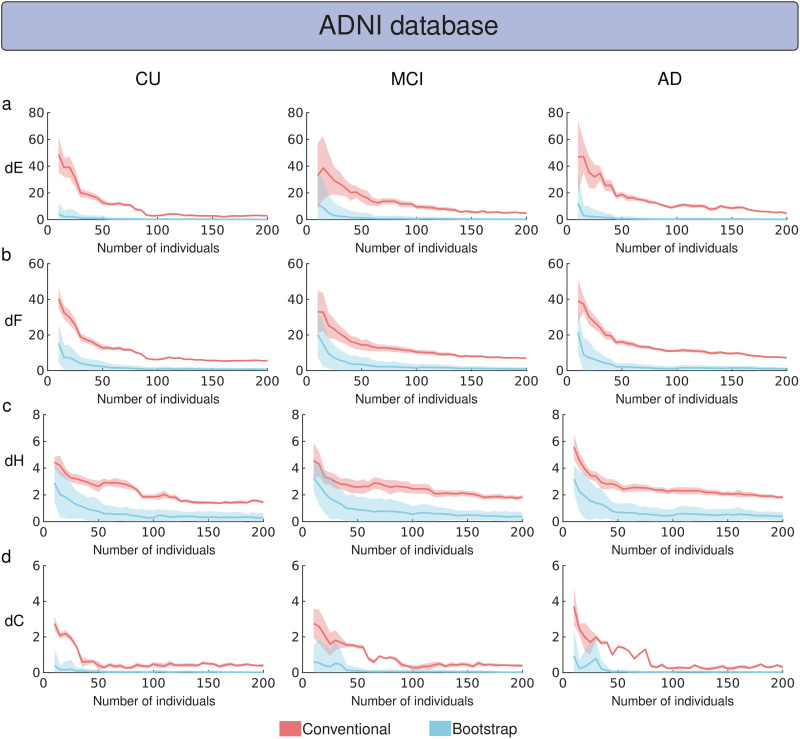
Comparison between the conventional and the MS bootstrap method as a function of the dataset size using the ADNI cohort. Groups CU, MCI, and AD were network outlier attacked 256 times (*P*_*o*_ = 2%) for each dataset size defined in the interval [10, 15, 20, …, 200]. MS bootstrap MBNs were constructed using the mean matrix criterion, *α* = 0.0001, and *n* was set to 9,300. The stability of MBNs was evaluated using dE, dF, dH, and dC, shown in panels (A), (B), (C), and (D), respectively, for both conventional and bootstrap methods. Bold lines represent the mean stability measure values, while the light shadows indicate three times the standard deviation from the mean.

**Figure 8.**
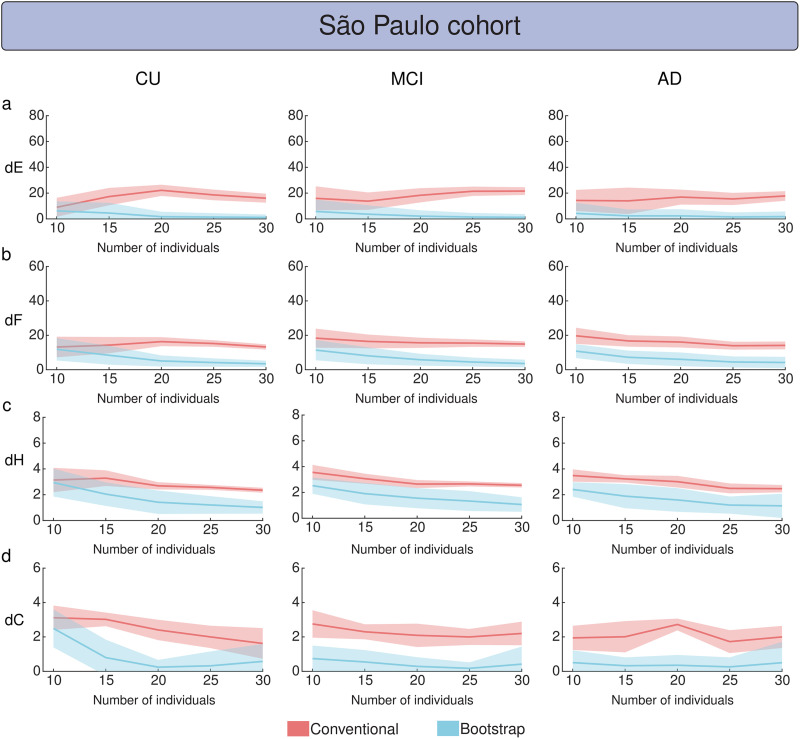
Comparison between the conventional and the MS bootstrap method as a function of the dataset size using the FMUSP cohort. Groups CU, MCI, and AD were network outlier attacked 256 times (*P*_*o*_ = 2%) for each dataset size defined in the interval [10, 15, 20, …, 200]. MS bootstrap MBNs were constructed using the mean matrix criterion, *α* = 0.0001, and *n* was set to 9,300. The stability of MBNs was evaluated using dE, dF, dH, and dC, shown in panels (A), (B), (C), and (D), respectively, for both conventional and bootstrap methods. Bold lines represent the mean stability measure values, while the light shadows indicate three times the standard deviation from the mean.

The evaluation of MBN stability (with 2% of outlier attacks) as a function of dataset size using the ADNI cohort (i.e., the number of individuals used to construct MBNs) revealed that the MS bootstrap scheme generated greater stability in groups with fewer individuals (Figure 7A–D). Specifically, the conventional method generally requires 100 to 200 samples to achieve network stability, while the MS scheme reaches stability with fewer samples, typically between 50 and 100 (Figure 7A–D). In summary, average network stability measures (dE, dF, dH, and dC) computed for the MS bootstrap scheme converged faster to their minimum stability values than the conventional construction method. Similarly, the MS bootstrap scheme proved to be more stable against network outlier attacks with 5% and 8% outliers, depending on the dataset size (Supporting Information Figures S8 and S9). Regarding the dataset size issue, the subsampling scheme was more stable than the conventional method against network outlier attacks with 2%, 5%, and 8% of outliers (Supporting Information Figures S10–S12) as a function of the dataset size. We also used the FMUSP cohort to evaluate MBN stability, since it contains fewer individuals (Figure 8A–D). The MS bootstrap scheme showed better stability performance, requiring as few as 20 samples to provide stable networks compared with the conventional method.

## DISCUSSION

We proposed a novel MS scheme to construct stable MBNs, indexed by an innovative stability strategy. By applying bootstrap sampling to a dataset of static [^18^F]FDG-PET images, we demonstrated that it is possible to select a representative network from multiple assembled MBNs that preserve the group’s characteristics and overall organization. Other frameworks employing dynamic [^18^F]FDG-PET simultaneously acquired with MRI in hybrid systems have recently been proposed (Deery et al., 2024; Hahn et al., 2018; Reed et al., 2023; Volpi et al., 2023). However, their application remains limited due to the predominance of static [^18^F]FDG-PET acquisitions in currently available datasets. Additionally, the generated MBN showed greater resilience to outlier attacks. We introduced a novel thresholding method that leverages the intersubject variability of the MBNs’ degree distribution to retain only the connections that are highly probable among group subjects. Evaluating MBN stability is challenging due to the absence of a known ground truth, making the definition of a stability criterion inherently difficult. In this report, we circumvented the issue of defining a unique MBN ground truth for CU, MCI, and AD groups by expanding on the concept of node attacks (Albert et al., 2000) and introducing an outlier attack model to perturb groups of [^18^F]FDG-PET data, similar to the approach in Meinshausen and Bühlmann (2010). This strategy was inspired by previous work (Rodrigues et al., 2018) and aligns with the concept of differential privacy (Dwork et al., 2015), which holds that a small number of data samples should not significantly affect an algorithm’s output. Using the framework proposed by Soundarajan and colleagues, we addressed the problem of measuring the MBN’s stability by evaluating the similarity between a group representative network and its correspondent perturbed version (Soundarajan et al., 2014). We tested our method using traditional similarity metrics in the field of pattern recognition. More specifically, to compare the general similarity between adjacency matrices, we have used the Frobenius norm and the dH. Additionally, to compare the characteristics of the MBNs, we computed graph theoretical measures and compared the representative MBN with its perturbed version in the feature space defined by these graph measures. We used the dE and the dC to assess similarity, with smaller values indicating greater stability of the MBN.

Notably, we have not found statistically significant differences in the stability measures between the mean, mode, and median construction criteria. This indicates that the constructed MBNs may be Gaussian distributed, forming a cluster in a high dimensional manifold. Under this view, identifying the group representative MBN is equivalent to finding the centroid of this Gaussian-shaped cluster, which explains why there are no significant differences in the stability measures between these criteria. It is worth noting that the proposed MS scheme does not rely specifically on the connectivity measure of choice. Here, for simplicity, we have used the Pearson correlation measure—which is widely used for constructing MBNs in brain PET imaging studies—to introduce the overall formalism of this approach. Although we used Pearson correlation and tested different thresholding strategies (FDR, Bonferroni, and Pmap), the MS scheme could be easily adapted to embrace other types of measures and correction methods, such as partial and Spearman correlations, as well as measures more sensible to nonlinear interactions such as mutual information and the maximal information coefficient and other relative thresholding methods using *Z*-score, percentile-based, or histogram-based metrics (Reshef et al., 2011). Similarly, the strategy employed for stability evaluation can be applied regardless of how the adjacency matrices (i.e., the MBNs) are organized, accommodating different choices of the connectivity measure. Here, we did not fully explore the vast domain of available similarity measures between matrices in the literature. The stability results obtained with the MS scheme (see Figures 5–8) revealed a great potential for assembling stable MBNs, that is, MBNs assembled with the proposed scheme were significantly more stable than the conventional method. Additional similarity measures could potentially add information but were not tested here (Koutroumbas & Theodoridis, 2008).

The results also demonstrated that the MS scheme is robust to the data imbalance issue, showing low variability between the CU, MCI, and AD groups across different scenarios of group imbalance (ADASYN, Undersampled, and Imbalanced). Indeed, the overall dispersion of each group is smaller across imbalanced scenarios for the bootstrap MS scheme when compared with the conventional method. One could argue that the MS scheme may be a proper method for studying imbalanced datasets from large-scale clinical trials, such as the ADNI cohort. Furthermore, our findings highlight the potential of the bootstrap MS scheme to reduce the number of individuals required to assemble group representative MBNs with enhanced stability, which could make MBNs a more widely used secondary outcome in brain PET studies. The computational burden of the bootstrap approach may be significant in some cases, especially in large-scale studies or when computational resources are limited. Optimizing the sampling strategy or considering parallel processing methods could help alleviate this issue and improve computational efficiency. The ADNI and FMUSP data were processed and analyzed independently using different tools. Nevertheless, the results are similar, which suggests that the method does not depend on the imaging processing pipeline. In terms of network patterns, we replicated prior findings demonstrating that MCI and AD brains exhibit a reduction in global metabolic connectivity (Arnemann et al., 2017; S.-Y. Huang et al., 2020; Kuang et al., 2019; Pagani et al., 2017). Our approach successfully captures meaningful biological results using a smaller number of participants, replicating findings from previous studies that used larger sample sizes. This method paves the way for future studies involving smaller cohorts, enabling the discovery of novel biological insights. The definition and characterization of metabolic networks remain an area of active debate (Sala et al., 2023; Vogel et al., 2023). These findings highlight the need for methodological frameworks that account for the unique properties of metabolic imaging, particularly regarding the relative merits of intra- versus intersubject approaches. Here, the MS scheme could offer additional insights into the definition and stability of metabolic networks, particularly in the context of group variability. Future applications of these approaches may help refine metabolic network analysis and improve their integration with other neuroimaging modalities. Future applications of these approaches may help refine metabolic network analysis and improve their integration with other neuroimaging modalities. In addition, the MS scheme is application-independent, making it suitable for studying various brain diseases using different measures. It also offers the flexibility to assemble brain networks derived from multiple PET radiotracers. Moreover, since the proposed scheme does not depend on data-specific parameters, it could potentially be applied to other mammalian species, such as nonhuman primates and rodents.

## CONCLUSION

In summary, in the present study, we provided a straightforward method to assemble reliable brain networks with multiple applications in brain PET imaging research. Our method has the potential to considerably increase PET data reutilization and advance our understating of brain network dynamics in health and disease.

## ACKNOWLEDGMENTS

Data collection and sharing for this project was funded by the Alzheimer’s Disease Neuroimaging Initiative (ADNI) (National Institutes of Health Grant U01 AG024904) and DOD ADNI (Department of Defense Award Number W81XWH-12-2-0012). ADNI is funded by the National Institute on Aging, the National Institute of Biomedical Imaging and Bioengineering, and through generous contributions from the following: AbbVie, Alzheimer’s Association; Alzheimer’s Drug Discovery Foundation; Araclon Biotech; BioClinica, Inc.; Biogen; Bristol-Myers Squibb Company; CereSpir, Inc.; Cogstate; Eisai Inc.; Elan Pharmaceuticals, Inc.; Eli Lilly and Company; EuroImmun; F. Hoffmann-La Roche Ltd. and its affiliated company Genentech, Inc.; Fujirebio; GE Healthcare; IXICO Ltd.; Janssen Alzheimer Immunotherapy Research & Development, LLC.; Johnson & Johnson Pharmaceutical Research & Development LLC.; Lumosity; Lundbeck; Merck & Co., Inc.; Meso Scale Diagnostics, LLC.; NeuroRx Research; Neurotrack Technologies; Novartis Pharmaceuticals Corporation; Pfizer Inc.; Piramal Imaging; Servier; Takeda Pharmaceutical Company; and Transition Therapeutics. The Canadian Institutes of Health Research is providing funds to support ADNI clinical sites in Canada. Private sector contributions are facilitated by the Foundation for the National Institutes of Health (www.fnih.org). The grantee organization is the Northern California Institute for Research and Education, and the study is coordinated by the Alzheimer’s Therapeutic Research Institute at the University of Southern California. ADNI data are disseminated by the Laboratory for Neuro Imaging at the University of Southern California.

## SUPPORTING INFORMATION

Supporting information for this article is available at https://doi.org/10.1162/NETN.a.23.

## AUTHOR CONTRIBUTIONS

Guilherme Schu: Conceptualization; Data curation; Formal analysis; Methodology; Validation; Visualization; Writing – original draft. Christian Limberger: Data curation; Formal analysis; Methodology; Validation; Visualization; Writing – original draft. Wagner S. Brum: Data curation; Formal analysis; Methodology. Marco Antônio De Bastiani: Data curation; Formal analysis; Methodology; Writing – review & editing. Yuri Elias Rodrigues: Data curation; Formal analysis; Methodology. Julio Cesar de Azeredo: Data curation; Formal analysis; Methodology. Tharick Ali Pascoal: Funding acquisition; Project administration; Resources; Supervision; Writing – review & editing. Andrea Lessa Benedet: Writing – review & editing. Sulantha Mathotaarachchi: Writing – review & editing. Pedro Rosa-Neto: Funding acquisition; Project administration; Resources; Supervision; Writing – review & editing. Jorge Almeida: Resources; Supervision; Writing – review & editing. Daniele de Paula Faria: Data curation; Formal analysis; Methodology; Writing – review & editing. Fábio Luiz de Souza Duran: Data curation; Formal analysis; Methodology; Writing – review & editing. Carlos Alberto Buchpiguel: Data curation; Formal analysis; Methodology; Writing – review & editing. Artur Martins Coutinho: Data curation; Formal analysis; Methodology; Writing – review & editing. Geraldo F. Busatto: Data curation; Formal analysis; Methodology; Writing – review & editing. Eduardo R. Zimmer: Conceptualization; Data curation; Formal analysis; Methodology; Validation; Visualization; Writing – original draft.

## DATA AND CODE AVAILABILITY

Human data used in this study were downloaded from the Alzheimer’s Disease Neuroimaging Initiative database (adni.loni.usc.edu). Metabolic data used to generate the MBNs and conduct the experiments may be available from the corresponding author upon request. For additional information about the FMUSP cohort, contact Dr. Geraldo F. Busatto (geraldo.busatto@hc.fm.usp.br). The implementation codes are available at https://github.com/guischu09/MultipleSampling-MBN.

## FUNDING INFORMATION

Jorge Almeida, European Research Council (ERC) under the European Union’s Horizon 2020 research and innovation program, ERC Starting Grant “ContentMAP” (https://cordis.europa.eu/project/id/802553/results), Award ID: 802553. Jorge Almeida, European Research Executive Agency Widening program under the European Union’s Horizon Europe Grant “CogBooster” (https://cordis.europa.eu/project/id/101087584), Award ID: 101087584. Eduardo Rigon Zimmer, Conselho Nacional de Desenvolvimento Científico e Tecnológico (https://dx.doi.org/10.13039/501100003593), Award ID: 312410/2018-2. Eduardo Rigon Zimmer, Conselho Nacional de Desenvolvimento Científico e Tecnológico (https://dx.doi.org/10.13039/501100003593), Award ID: 435642/2018-9. Eduardo Rigon Zimmer, Conselho Nacional de Desenvolvimento Científico e Tecnológico (https://dx.doi.org/10.13039/501100003593), Award ID: 312306/2021-0. Eduardo Rigon Zimmer, Conselho Nacional de Desenvolvimento Científico e Tecnológico (https://dx.doi.org/10.13039/501100003593), Award ID: 409066/2022-2. Eduardo Rigon Zimmer, Conselho Nacional de Desenvolvimento Científico e Tecnológico (https://dx.doi.org/10.13039/501100003593), Award ID: 409595/2023-3. Eduardo Rigon Zimmer, Fundação de Amparo à Pesquisa do Estado do Rio Grande do Sul (https://dx.doi.org/10.13039/501100004263), Award ID: 21/2551-0000673-0. Eduardo Rigon Zimmer, Alzheimer’s Association (https://dx.doi.org/10.13039/100000957), Award ID: AARGD-21-850670. Eduardo Rigon Zimmer, Conselho Nacional de Desenvolvimento Científico e Tecnológico (https://dx.doi.org/10.13039/501100003593), Award ID: 16/2551-0000475-7. Eduardo Rigon Zimmer, Instituto Nacional de Ciência e Tecnologia para Excitotoxicidade e Neuroproteção (https://dx.doi.org/10.13039/501100007395), Award ID: 465671/2014-4. Eduardo Rigon Zimmer, Instituto Serrapilheira (https://dx.doi.org/10.13039/501100013275), Award ID: Serra-1912-31365. Eduardo Rigon Zimmer, Alzheimer’s Association (https://dx.doi.org/10.13039/100000957), Award ID: ALZNAN-22-928381. Daniele de Paula Faria, Fundação de Amparo à Pesquisa do Estado de São Paulo (https://dx.doi.org/10.13039/501100001807), Award ID: 2012/50239-6. Marco Antônio De Bastiani, Alzheimer’s Association (https://dx.doi.org/10.13039/100000957), Award ID: AARFD-23-1148735. Geraldo F. Busatto, Fundação de Amparo à Pesquisa do Estado de São Paulo (https://dx.doi.org/10.13039/501100001807), Award ID: 2012/50239-6.

## CONFLICT OF INTEREST

E.R. Zimmer has served on the scientific advisory board, as a consultant or speaker for Nintx, Novo Nordisk, Biogen, Lilly, Magdalena Biosciences and masima. A.M.C., M.A.B., and E.R.Z. are co-founders and shareholders of masima. The other authors declare that they have no competing interests.

## Supplementary Material



## TECHNICAL TERMS

[^18^F]Fluorodeoxyglucose positron emission tomography ([^18^F]FDG-PET): Brain imaging technique measuring brain glucose metabolism using a radioactive glucose analog, useful for detecting or staging neurodegenerative diseases.
Standardized uptake value ratio (SUVR): A semiquantitative PET metric comparing radiotracer uptake in target regions with a reference region for standardization.
Bootstrap: A resampling method that estimates statistics’ variability by repeatedly sampling with replacement from the observed dataset.
Global efficiency: A graph theory metric measuring how efficiently information is exchanged across the whole brain network.
Assortativity coefficient: Quantifies a network’s tendency for nodes to connect with others that have similar properties.
Average degree: The mean number of connections per node in a network, reflecting overall network connectivity.
Average strength: The mean sum of edge weights per node, indicating the overall intensity of connections in a weighted network.
Density: Proportion of actual connections to all possible connections in a network, representing how connected the network is.
Average clustering coefficient: Measures how nodes tend to form local clusters, indicating segregation or local efficiency in a brain network.
Adaptive synthetic sampling approach for imbalanced learning (ADASYN): Machine learning technique generating synthetic minority class samples to improve classifier performance on imbalanced datasets.
