# Supplementary material for: Stable brain PET metabolic networks using a multiple sampling scheme
Source: Netw Neurosci. 2025 Sep 19;9(3):1087–109. doi: 10.1162/NETN.a.23 (PMC12548669; doi:10.1162/NETN.a.23)
Supplement: Supplementary file 1 [file netn-9-3-1087-s001.pdf]

Supplemental materials

Stable brain PET metabolic networks using a multiple sampling scheme

Guilherme Schu[1,2,*], Christian Limberger[3,*], Wagner S. Brum[3], Marco Antônio De Bastiani[4], Yuri Elias Rodrigues[5], Julio Cesar de Azeredo[3], Tharick A. Pascoal[6], Andrea Lessa Benedet[7], Sulantha Mathotaarachchi[8], Pedro Rosa-Neto[8,9,10], Jorge Almeida[11,12], Daniele de Paula Faria[13,14], Fábio Luiz de Souza Duran[14,15], Carlos Alberto Buchpiguel[13,14], Artur Martins Coutinho[13,14,16], Geraldo F Busatto[14,15], and Eduardo R. Zimmer[3,4,8,17,18,#] for the Alzheimer's Disease Neuroimaging Initiative[&]

[1]Idiap Research Institute, Martigny, Switzerland.

[2]École Polytechnique Fédérale de Lausanne, Lausanne, Switzerland.

[3]Graduate Program in Biological Sciences: Biochemistry, Universidade Federal do Rio Grande do Sul (UFRGS), Porto Alegre, Brazil.

[4]Graduate Program in Biological Sciences: Pharmacology and Therapeutics, UFRGS, Porto Alegre, Brazil.

[5]Strathclyde Institute of Pharmacy and Biomedical Sciences, University of Strathclyde, Glasgow, Scotland.

[6]Department of Psychiatry and Department of Neurology, University of Pittsburgh, Pittsburgh, PA, USA.

[7]Department of Psychiatry and Neurochemistry, The Sahlgrenska Academy at the University of Gothenburg, Mölndal, Sweden.

[8]Translational Neuroimaging Laboratory, The McGill University Research Centre for Studies in Aging, Douglas Mental Health Institute, Montreal, QC H4H 1R3, Canada.

[9]Department of Neurology and Neurosurgery, McGill University, Montreal, QC H3A 0G4, Canada.

[10]Montreal Neurological Institute, Montreal, QC H3A 2B4, Canada.

[11]Proaction Laboratory, Faculty of Psychology and Education Sciences, University of Coimbra, Coimbra, Portugal.

[12]CINEICC, Faculty of Psychology and Education Sciences, University of Coimbra, Coimbra, Portugal.

[13]Laboratory of Nuclear Medicine (LIM 43), Department of Radiology and Oncology, Faculdade de Medicina FMUSP, Universidade de São Paulo, Sao Paulo, SP, Brazil.
[14]Núcleo de Apoio a Pesquisa em Neurociência Aplicada (NAPNA), Universidade de São Paulo, São Paulo, SP, Brazil.
[15]Laboratory of Psychiatric Neuroimaging (LIM 21), Department of Psychiatry, Faculdade de Medicina FMUSP, Universidade de São Paulo, Sao Paulo, SP, Brazil.
[16]Nuclear Medicine Service, Centro de Diagnosticos por Imagem, Hospital Sirio-Libanes, Sao Paulo, Brazil.
[17]Department of Pharmacology, UFRGS, Porto Alegre, Brazil.
[18]Brain Institute of Rio Grande do Sul, PUCRS, Porto Alegre, Brazil.
*Guilherme Schu and Christian Limberger contributed equally to this work.

[#]Corresponding author:

Eduardo R. Zimmer, PhD (E.R. Zimmer)
Department of Pharmacology, Universidade Federal do Rio Grande do Sul (UFRGS)
2600 Ramiro Barcelos street, 90035-003, Porto Alegre, RS, Brazil
Email address: eduardo.zimmer@ufrgs.br
Telephone: +55 51 33085558
Fax: +55 51 33085544
Website: www.zimmer-lab.org

[&]Data used in preparation of this article were obtained from the Alzheimer's Disease Neuroimaging Initiative (ADNI) database (adni.loni.usc.edu). As such, the investigators within the ADNI contributed to the design and implementation of ADNI and/or provided data but did not participate in analysis or writing of this report. A complete listing of ADNI investigators can be found at: http://adni.loni.usc.edu/wp-content/uploads/how_to_apply/ADNI_Acknowledgement_List.pdf

Mode group representative matrix computation

Previously we have used the mean matrix as the representative MBN of a group of interest. As an alternative, one could use the median or the mode criterion to decide which network should be classified as the group representative one. In order to compute the mode criterion, we have generalized the optimal Bayesian approach introduced by our previous work (Schu & Scharcanski, 2018). In this method, the representative network of the group is defined as the one located at the mode of the underlying distribution of the data. We assume that the posterior probability $p(M^{k,r}|\Phi)$ of network M^k to correspond to the group representative network M^r (i.e. $M^{k,r}$), given the set Φ, follows a mixture of matrix normal distributions:

$$p(M^{k,r}|\Phi) = \frac{\sum_{t=1}^{n} \mathcal{MN}(M^k \vee \overline{M}=M^t,U,V)\Pi_t}{\sum_{k=1}^{n}\sum_{t=1}^{n} \mathcal{MN}(M^k \vee \overline{M}=M^t,U,V)\Pi_t}, \quad \text{(S1)}$$

where $\overline{M} \in \mathbb{R}^{d\times d}$ is the mean, $U \in \mathbb{R}^{d\times d}$ and $V \in \mathbb{R}^{d\times d}$ correspond to the variances among-rows and among-columns, respectively, and the weighting component Π_t corresponds to the frequency of occurrence of matrix M^k in the underlying distribution spanned by the set Φ, such that $0 \leqslant \Pi_t \leqslant 1$ and $\sum_{t=1}^{n} \Pi_t = 1$.

In the mixture, each matrix normal distribution $\mathcal{MN}(M^k|M = M^t, U, V)$ is evaluated at matrix M^k and can be written as the equivalent multivariate Gaussian:

$$\mathcal{N}\left(\overrightarrow{(M^k)}\middle|\overrightarrow{(M^t)}, U \otimes V = \mathbb{I}\right) = \frac{1}{(2\pi)^{d^2/2}} \exp\left[-\frac{1}{2}\overrightarrow{(M^k - M^t)}^T\right] \quad \text{(S2)}$$

where $\overrightarrow{(.)}$ is the vectorization operation, $U \otimes V$ is the Kronecker product of matrices U and V, which we assume is the identity matrix $\mathbb{I} \in \mathbb{R}^{d^2\times d^2}$, and the superscript T denotes the transpose operation. Using equation equation S2, we can rewrite the posterior probability $p(M^{k,r} \vee \Phi)$ as:

$$p(M^{k,r}|\Phi) = \frac{\sum_{t=1}^{n} \mathcal{N}\left(\overrightarrow{(M^k)}\middle|\overrightarrow{(M^t)}, \mathbb{I}\right)\Pi_t}{\sum_{k=1}^{n}\sum_{t=1}^{n} \mathcal{N}\left(\overrightarrow{(M^k)}\middle|\overrightarrow{(M^t)}, \mathbb{I}\right)\Pi_t}. \quad \text{(S3)}$$

In equation S2, we estimate the density at vector $\overrightarrow{(M^k)}$ by computing a linear combination of symmetric shaped Gaussian distributions (with covariance $\mathbb{I}$) centered at each mean vector $\overrightarrow{(M^t)}$. For a more detailed discussion regarding the advantages of using the above formalism see (Schu & Scharcanski, 2018). We find the representative matrix $\mathcal{M}^{k,r}$ using the Maximum a *Posteriori* (*MAP*) approach as follows:

$$\mathcal{M}^{k,r} = \underset{1 \leq k \leq n}{\arg\max} \{p(M^{k,r}|\Phi)\}. \tag{S4}$$

With this formulation, we seek to find the representative network $\mathcal{M}^{k,r}$, that maximizes the posterior probabilities $pp(M^{k,r}|\Phi)$, and corresponds to the mode of the underlying distribution spanned by Φ. For a given M^k, one can compute the posterior probability $p(M^{k,r}|\Phi)$ of M^k being a representative matrix $\mathcal{M}^{k,r}$ using equation S3. Once the group representative network $\mathcal{M}^{k,r}$ is determined, the network is corrected for multiple comparisons using false discovery rate (FDR).

Construction of MBNs using multiple subsampling

Alternatively to using bootstrap sampling, we have also tested the use of a subsampling approach. The adopted formalism of the subsampling scheme is slightly different to the bootstrap scheme and it is described as follows: let $X \in \mathbb{R}^{N \times d}$ be the original dataset matrix containing PET measures (e.g. SUVr) of N subjects (rows), for d volumes of interest (columns). The multiple subsampling method consists of generating n subsamples $Y^1, \ldots, Y^n$ of X. We denote a general subsample as $Y^k \subseteq X$ (with $1 \leq k \leq n$), where $Y^k \in \mathbb{R}^{N(k) \times d}$ and $N(k)$ is the number of subjects used to generate that matrix. In this context, we denote each column of Y^k as a vector y_j^k (with $1 \leq j \leq d$).

Given the aforementioned notations, we can construct the adjacency matrix $M^k \in \mathbb{R}^{d\times d}$, associated with the dataset $X^{(k)}$, by computing the Pearson correlation coefficient (for $p, q = 1, \ldots, d$) as follows:

$$r_{p,q}^k = \frac{\sum_{i=1}^{N(k)} \left(y_{i,p}^k - \overline{y_p^k}\right)\left(y_{i,q}^k - \overline{y_q^k}\right)}{\sqrt{\sum_{i=1}^{N(k)} \left(y_{i,p}^k - \overline{y_p^k}\right)^2} \sqrt{\sum_{i=1}^{N(k)} \left(y_{i,q}^k - \overline{y_q^k}\right)^2}}, \tag{S5}$$

where $y_{i,p}^k$ and $y_{i,q}^k$ correspond to the i-th element of the vectors y_p^k and y_q^k, $\overline{y_p^k} = \frac{1}{N(k)} \sum_{i=1}^{N(k)} y_{i,p}^k$ and $\overline{y_q^k} = \frac{1}{N(k)} \sum_{i=1}^{N(k)} y_{i,q}^k$ are the mean values of the vectors y_p^k and y_q^k, respectively.

In practice, the subset Y^k of the original dataset X is obtained using a random sample percentage S. For each subsampling, we generate randomly a S value between the interval of a maximum percentage (S_{max}) and minimum percentage (S_{min}). Hence, the construction of MBNs using the multiple subsampling scheme firstly generates the percentage S of subjects that will be removed from the original dataset X (i.e. rows of matrix X) and after, determines which subjects will be randomly drawn from X to generate Y^k. The input parameters, S_{max}, S_{min} and the total amount of random subsamplings n were evaluated using the train set and are described in the next section.

Parameter tuning with a subsampling scheme

MBN construction using the multiple subsampling scheme relies on the choice for the parameters of the maximum percentage (S_{max}) and the minimum percentage (S_{min}), which are used to generate randomly a sample percentage S. Also, our proposed construction method requires as input the number of subsamples n which defines the number of different networks that will be constructed to later estimate the representative matrix of a given group of interest. In our search for optimizing these parameters, we have used the train set as described in Section

2.2. In our experiments, we have setup S_{max} ∈ [10%, 12%, …, 30%] and have fixed S_{min}= 0.5%. The adopted optimization scheme, at first, fixes S_{max}, and iteratively computes the Bhattacharyya distance between the normalized degree distribution (i.e. the normalized histogram of edges connecting paired nodes) of the networks generated with the multiple subsampling scheme with parameters n = k and n = k +100 (with k∈ [100, 200, …, 9900]. When all possible values of $n$are searched, then S_{max} is incremented and the procedure is repeated. Finally, the parameters that minimize the Bhattacharyya distance value, among all, are the optimal parameters to be chosen. In our experiments we found the optimal parameters $S_{max} = 10\%$ and $n = 9300$.

Supplemental Figures

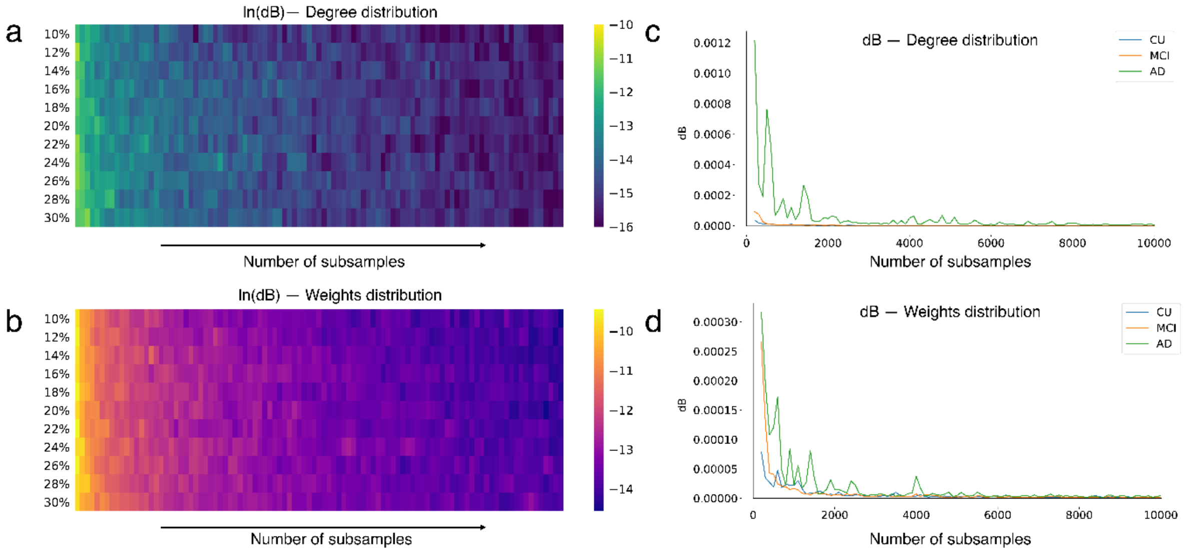

Supplemental Figure 1. Behavior of the Bhattacharrya distance (dB) as a function of the number of samples. The natural logarithm of dB values computed for the mean representative MBN degree distributions (a) and weight distributions (b) of MCI (i.e. the training set) as a function of the number of subsamples and S_{max}. Panels (c) and (d) show the behavior of dB values for the mean representative MBN degree and weight distributions, respectively, computed for CU, MCI, and AD (from the test set) as a function of the number of subsamples. Setup: $S_{max} = 10\%$.

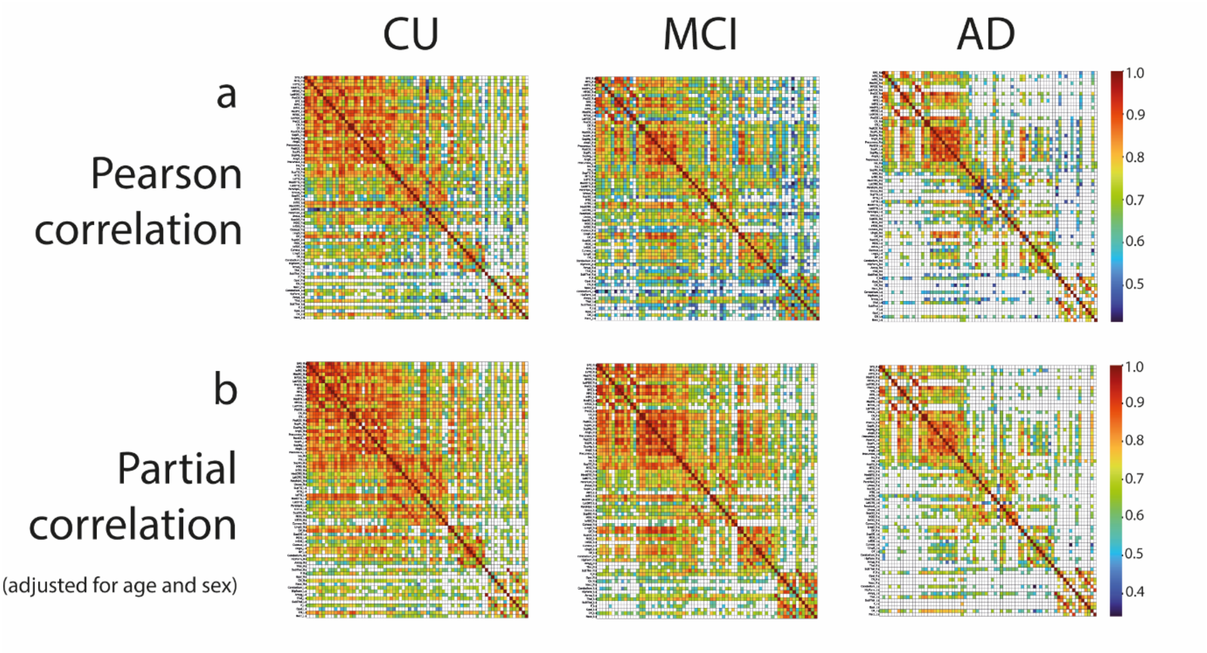

Supplemental Figure 2. Comparison between Pearson correlation and Partial correlation methods. The MBNs were computed for CU, MCI, and AD individuals from the ADNI cohort by using (a) Pearson correlation or (b) Partial correlation adjusted for covariates (age and sex). The adjacency matrices of correlation coefficients between brain regions present similar patterns when comparing both methods (uncorrected and corrected for age and sex).

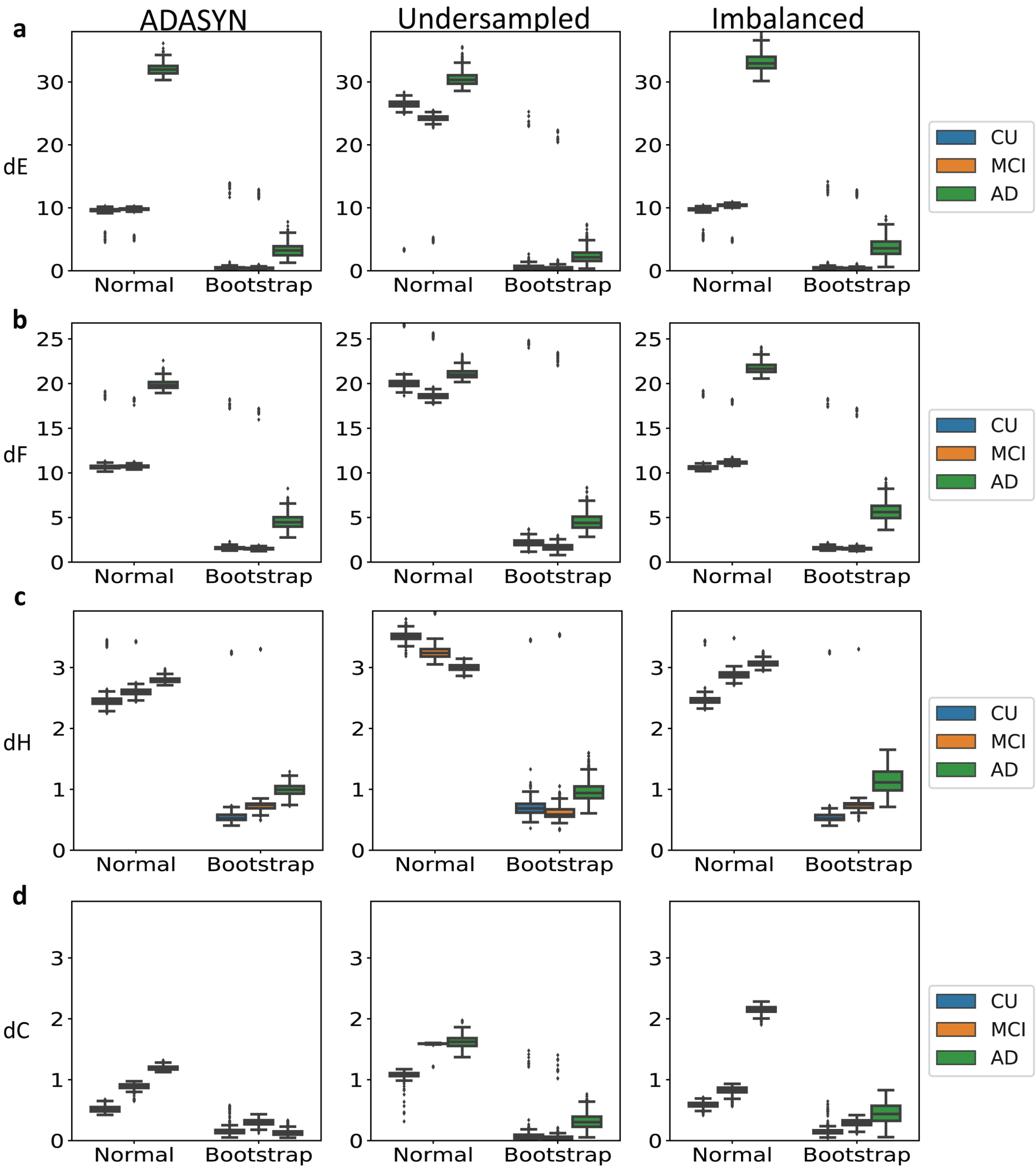

Supplemental Figure 3. Stability comparison between the conventional method and the MS bootstrap method under network outlier attacks with 5% outliers across different balance schemes. The stability of MBNs was compared between the conventional and MS bootstrap methods across different balance schemes. Groups CU, MCI, and AD were subjected to 256 network outlier attacks (P_o = 5%) under three balance schemes: ADASYN (left column), Undersampled (central column), and Imbalanced (right column). MS bootstrap MBNs were

generated using the mean matrix criterion with $\alpha = 0.0001$, and n set to 9,300. The stability of MBNs was evaluated using dE, dF, dH, and dC, shown in panels (a), (b), (c), and (d) for both conventional and bootstrap methods.

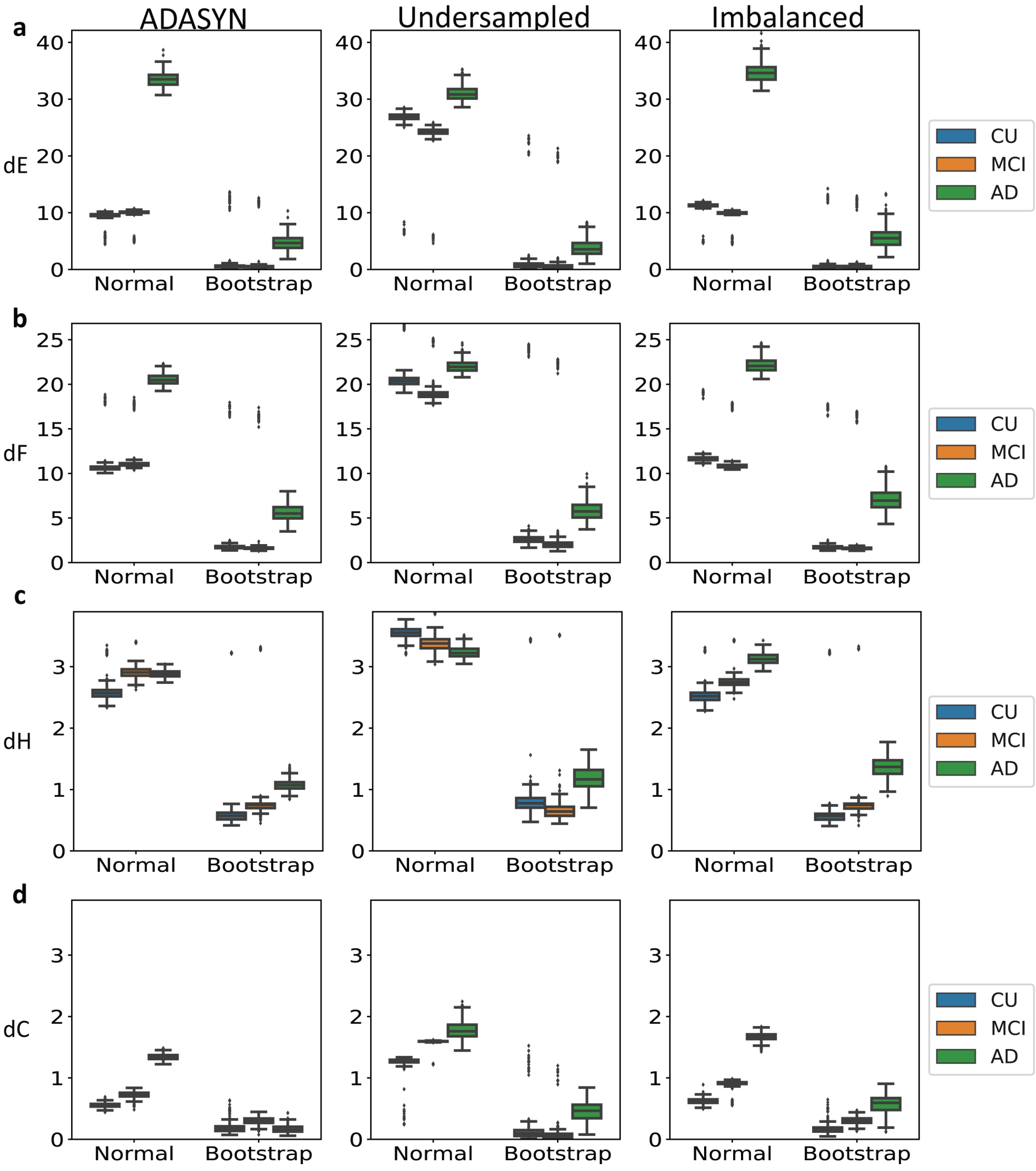

Supplemental Figure 4. Stability comparison between the conventional method and the MS bootstrap method under network outlier attacks with 8% outliers across different balance schemes. The stability of MBNs was compared between the conventional and MS bootstrap methods across different balance schemes. Groups CU, MCI, and AD were subjected to 256 network outlier attacks (P_o = 8%) under three balance schemes: ADASYN (left column), Undersampled (central column), and Imbalanced (right column). MS bootstrap MBNs were generated using the mean matrix criterion with α = 0.0001, and n set to 9,300. The stability of MBNs was evaluated using dE, dF, dH, and dC, shown in panels (a), (b), (c), and (d) for both conventional and bootstrap methods.

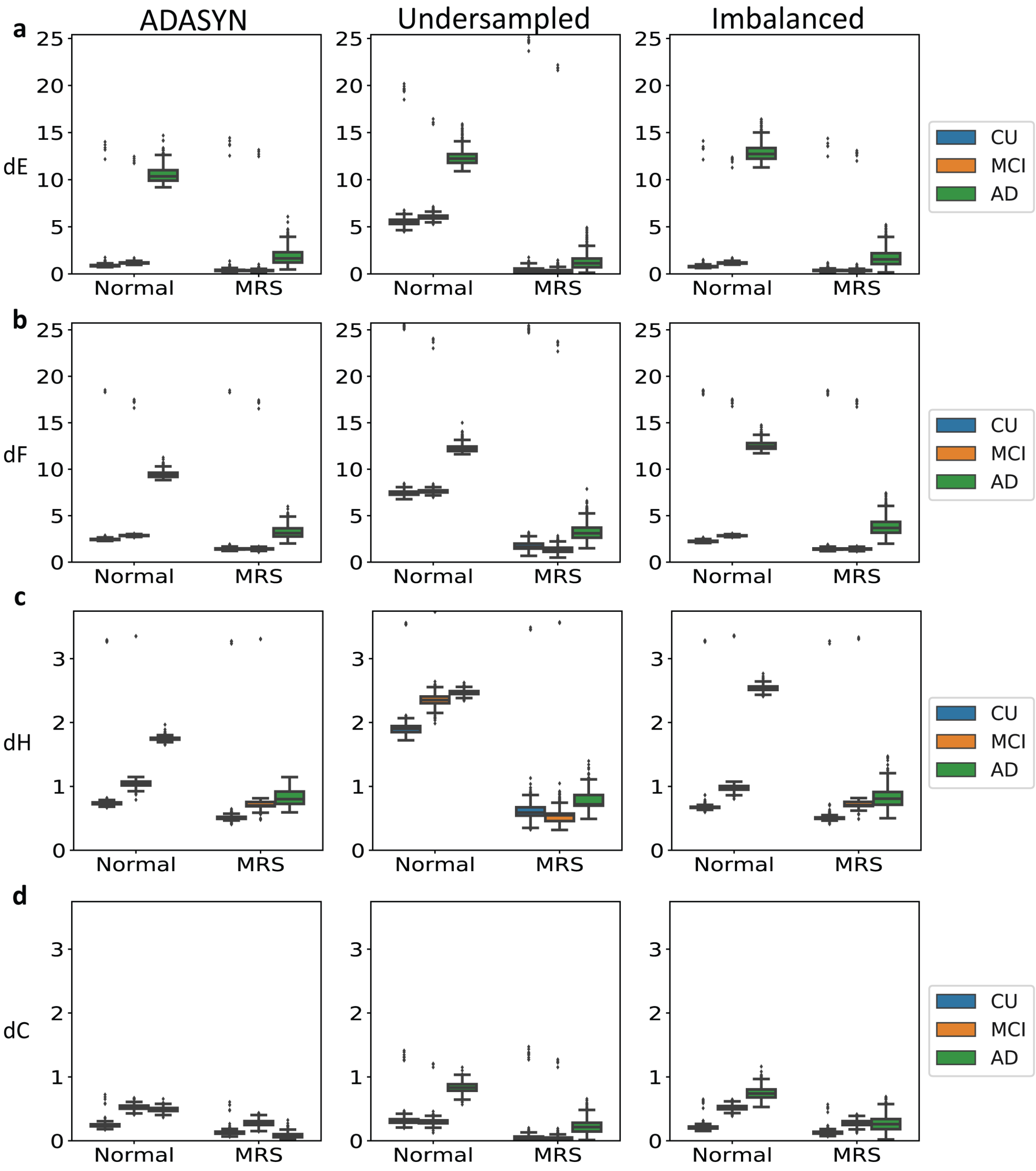

Supplemental Figure 5. Stability comparison between the conventional method and the multiple random subsampling (MRS) method under network outlier attacks with 2% outliers across different balance schemes. The stability of MBNs was compared between the conventional and MRS methods across different balance schemes. Groups CU, MCI, and AD were subjected to 256 network outlier attacks (P_o = 2%) under three balance schemes: ADASYN (left column), Undersampled (central column), and Imbalanced (right column). MS

bootstrap MBNs were generated using the mean matrix criterion with $\alpha = 0.0001$, and n set to 9,300. The stability of MBNs was evaluated using dE, dF, dH, and dC, shown in panels (a), (b), (c), and (d) for both conventional and bootstrap methods.

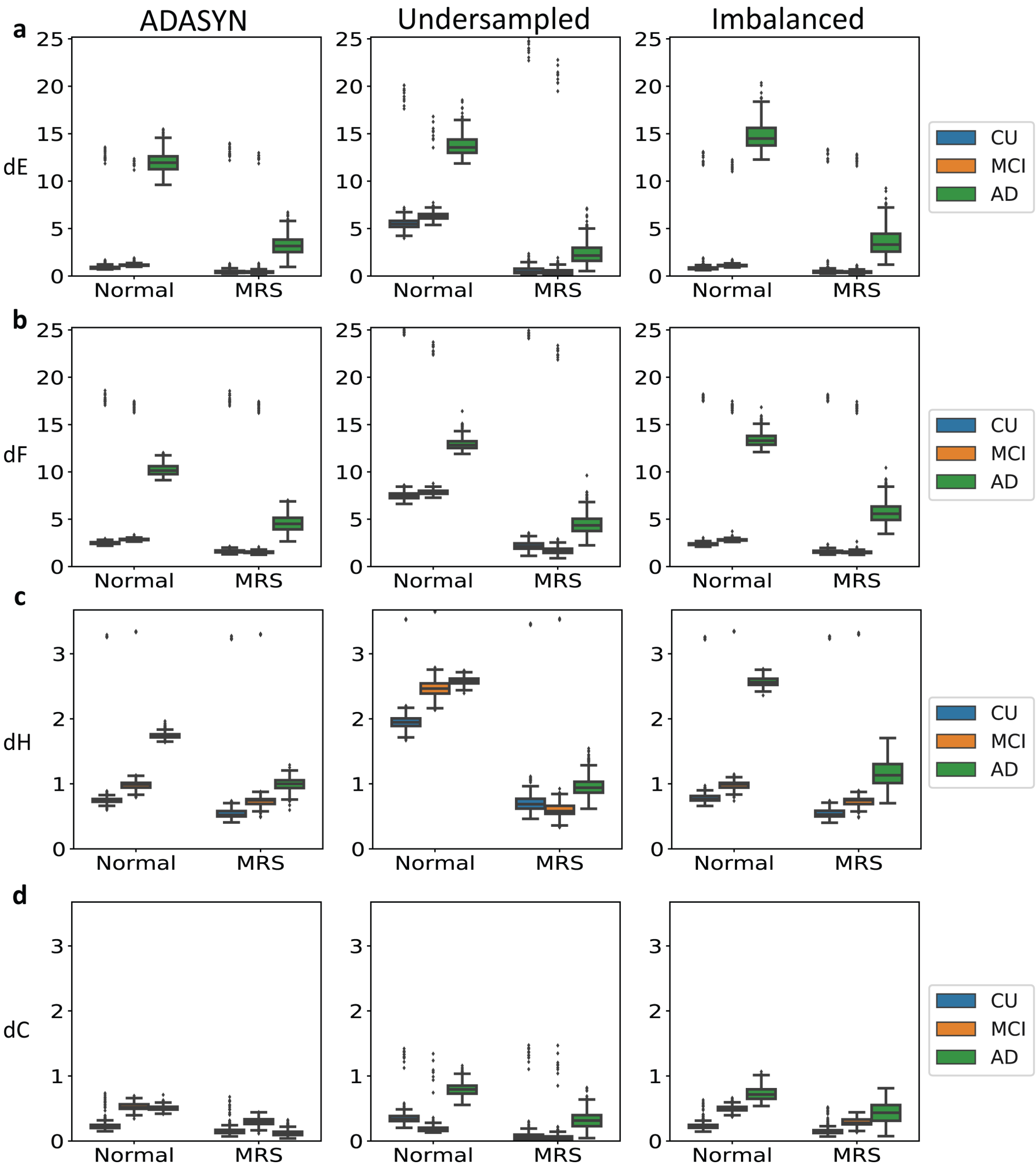

Supplemental Figure 6. Stability comparison between the conventional method and the multiple random subsampling (MRS) method under network outlier attacks with 5% outliers across different balance schemes. The stability of MBNs was compared between the conventional and MRS methods across different balance schemes. Groups CU, MCI, and AD were subjected to 256 network outlier attacks (P_o = 5%) under three balance schemes: ADASYN (left column), Undersampled (central column), and Imbalanced (right column). MS bootstrap MBNs were generated using the mean matrix criterion with α = 0.0001, and n set to 9,300. The stability of MBNs was evaluated using dE, dF, dH, and dC, shown in panels (a), (b), (c), and (d) for both conventional and bootstrap methods.

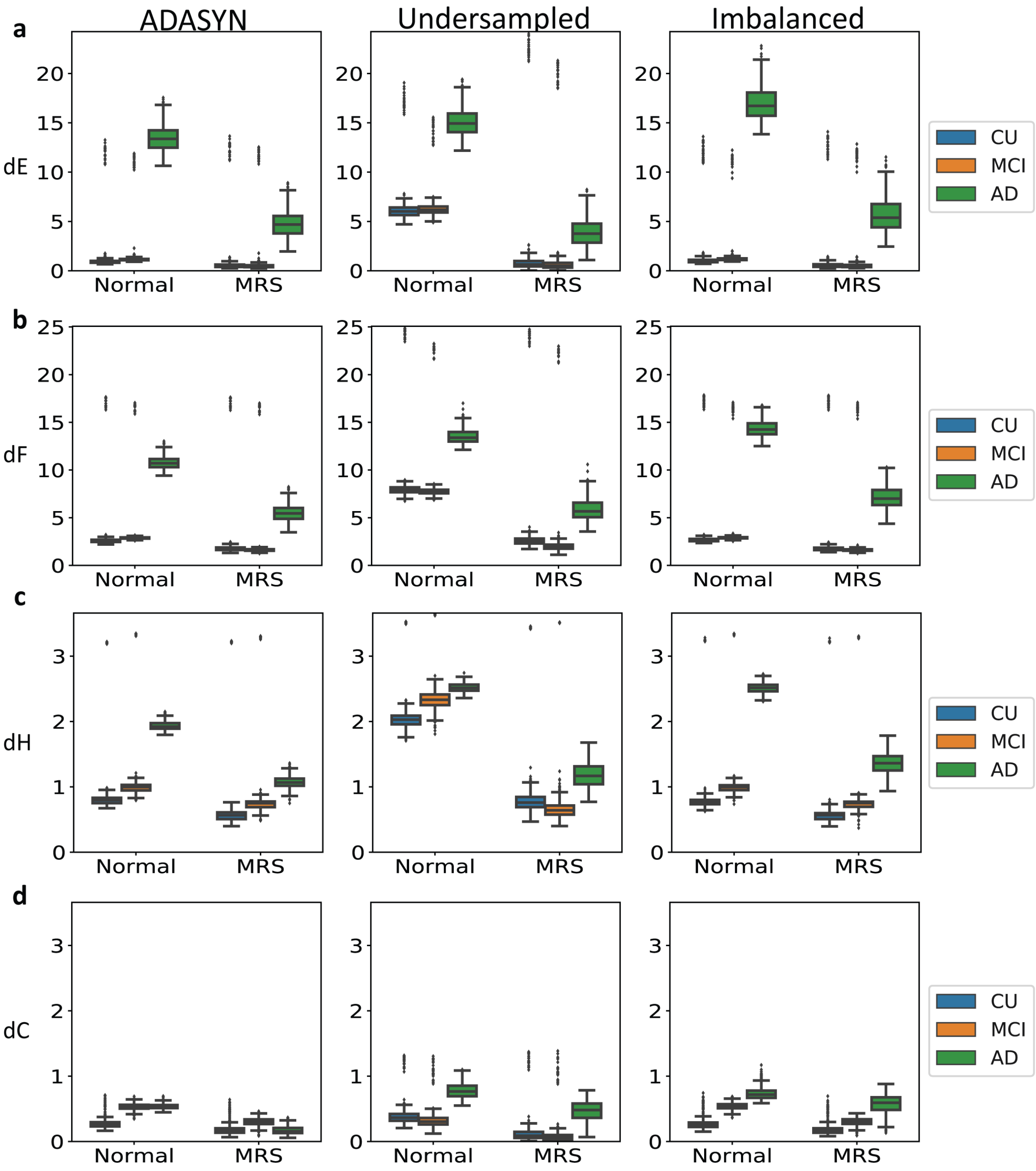

Supplemental Figure 7. Stability comparison between the conventional method and the multiple random subsampling (MRS) method under network outlier attacks with 8% outliers across different balance schemes. The stability of MBNs was compared between the conventional and MRS methods across different balance schemes. Groups CU, MCI, and AD were subjected to 256 network outlier attacks (P_o = 8%) under three balance schemes: ADASYN (left column), Undersampled (central column), and Imbalanced (right column). MS

bootstrap MBNs were generated using the mean matrix criterion with $\alpha = 0.0001$, and n set to 9,300. The stability of MBNs was evaluated using dE, dF, dH, and dC, shown in panels (a), (b), (c), and (d) for both conventional and bootstrap methods.

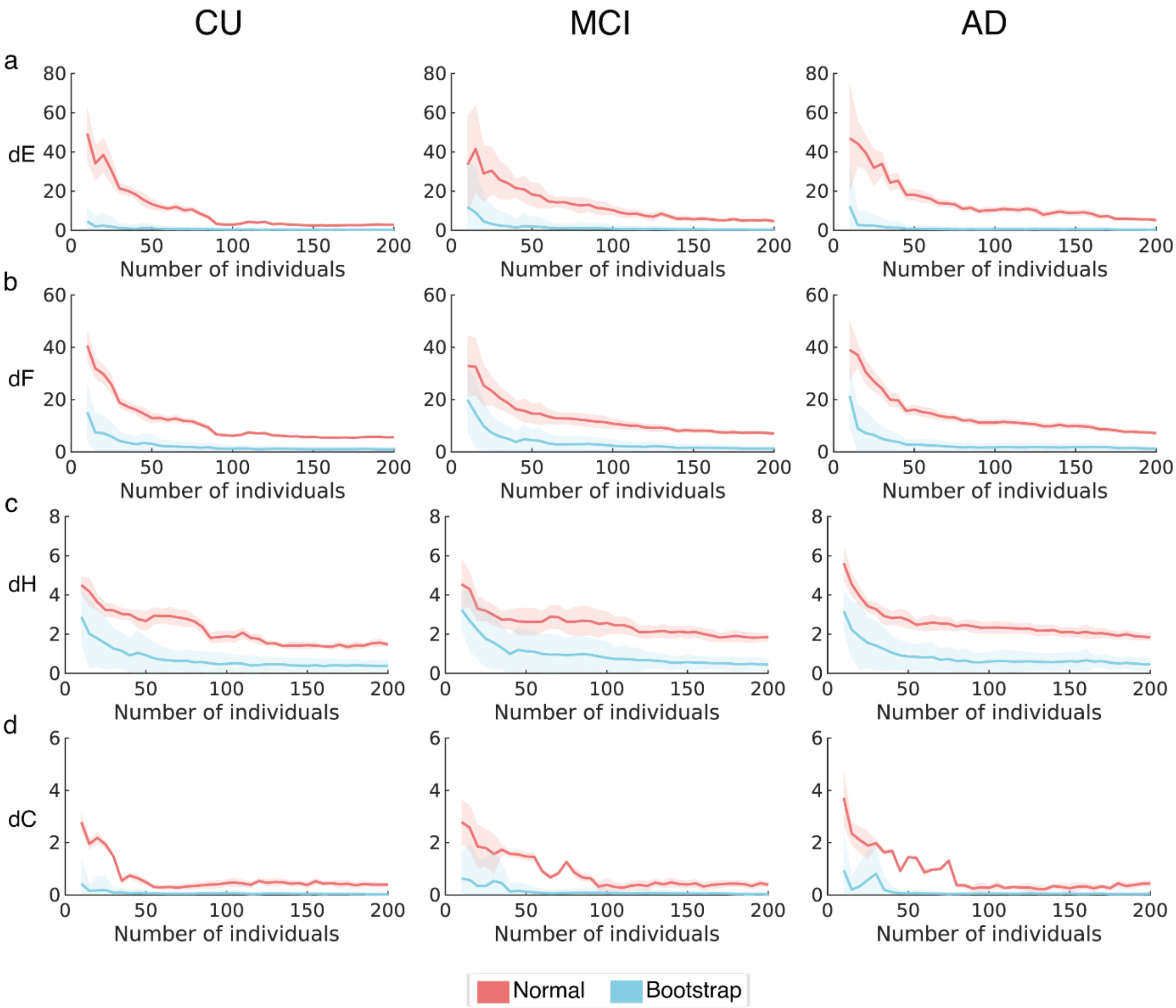

Supplemental Figure 8. Comparison between the conventional and the MS bootstrap method as a function of the dataset size and 5% outlier attack. Groups CU, MCI, and AD were network outlier attacked 256 times (P_o = 5%) for each dataset size defined in the interval [10, 15, 20, …, 200]. MS bootstrap MBNs were constructed using the mean matrix criterion, $\alpha = 0.0001$, and n was set to 9,300. The stability of MBNs was evaluated using dE, dF, dH,

and dC, shown in panels (a), (b), (c), and (d) for both conventional and bootstrap methods. Bold lines represent the mean stability measure values, while the light shadows indicate three times the standard deviation from the mean.

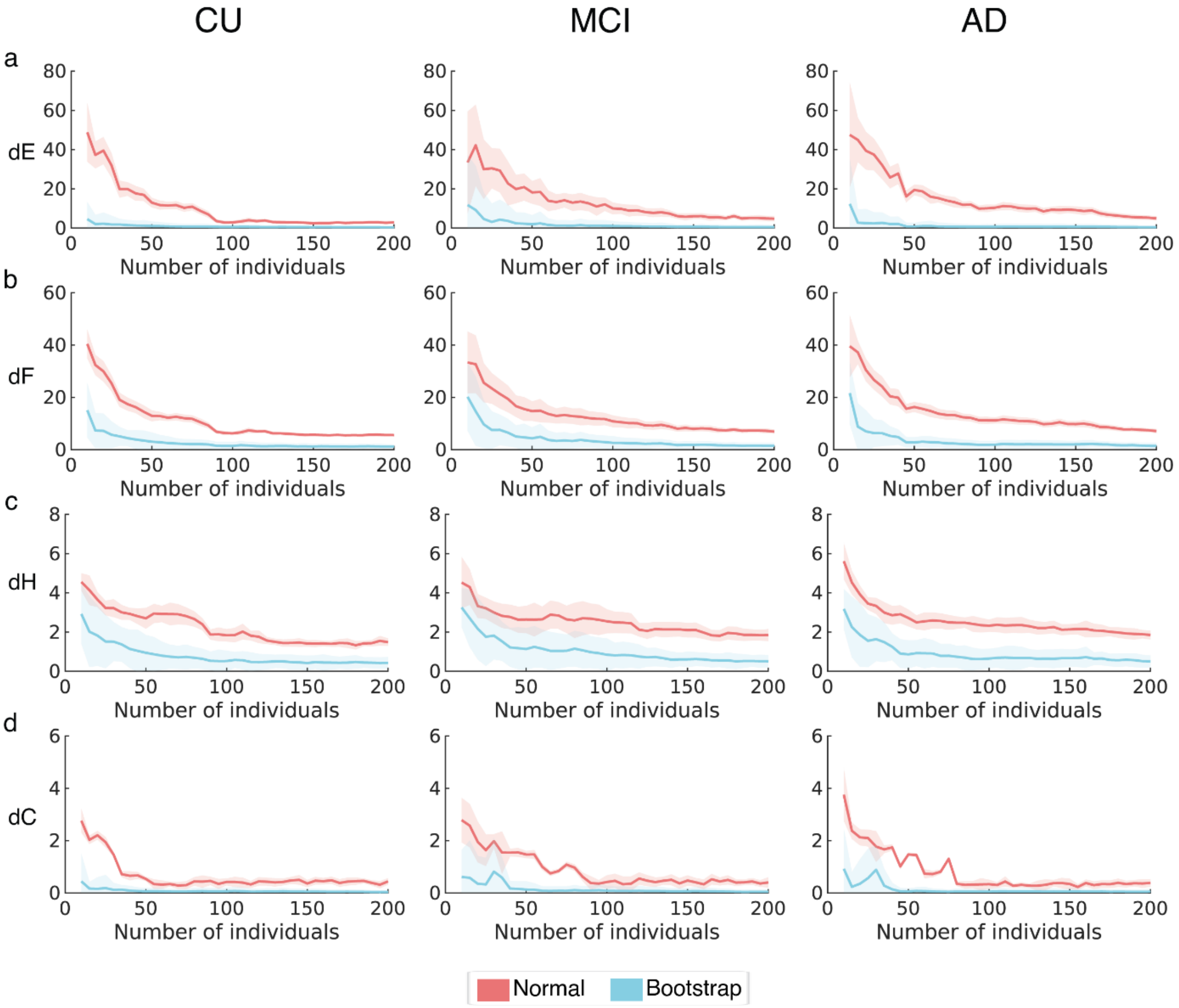

Supplemental Figure 9. Comparison between the conventional and the MS bootstrap method as a function of the dataset size and 8% outlier attack. Groups CU, MCI, and AD were network outlier attacked 256 times (P_o = 8%) for each dataset size defined in the interval [10, 15, 20, …, 200]. MS bootstrap MBNs were constructed using the mean matrix criterion, α = 0.0001, and n was set to 9,300. The stability of MBNs was evaluated using dE, dF, dH, and dC, shown in panels (a), (b), (c), and (d) for both conventional and bootstrap methods.

Bold lines represent the mean stability measure values, while the light shadows indicate three times the standard deviation from the mean.

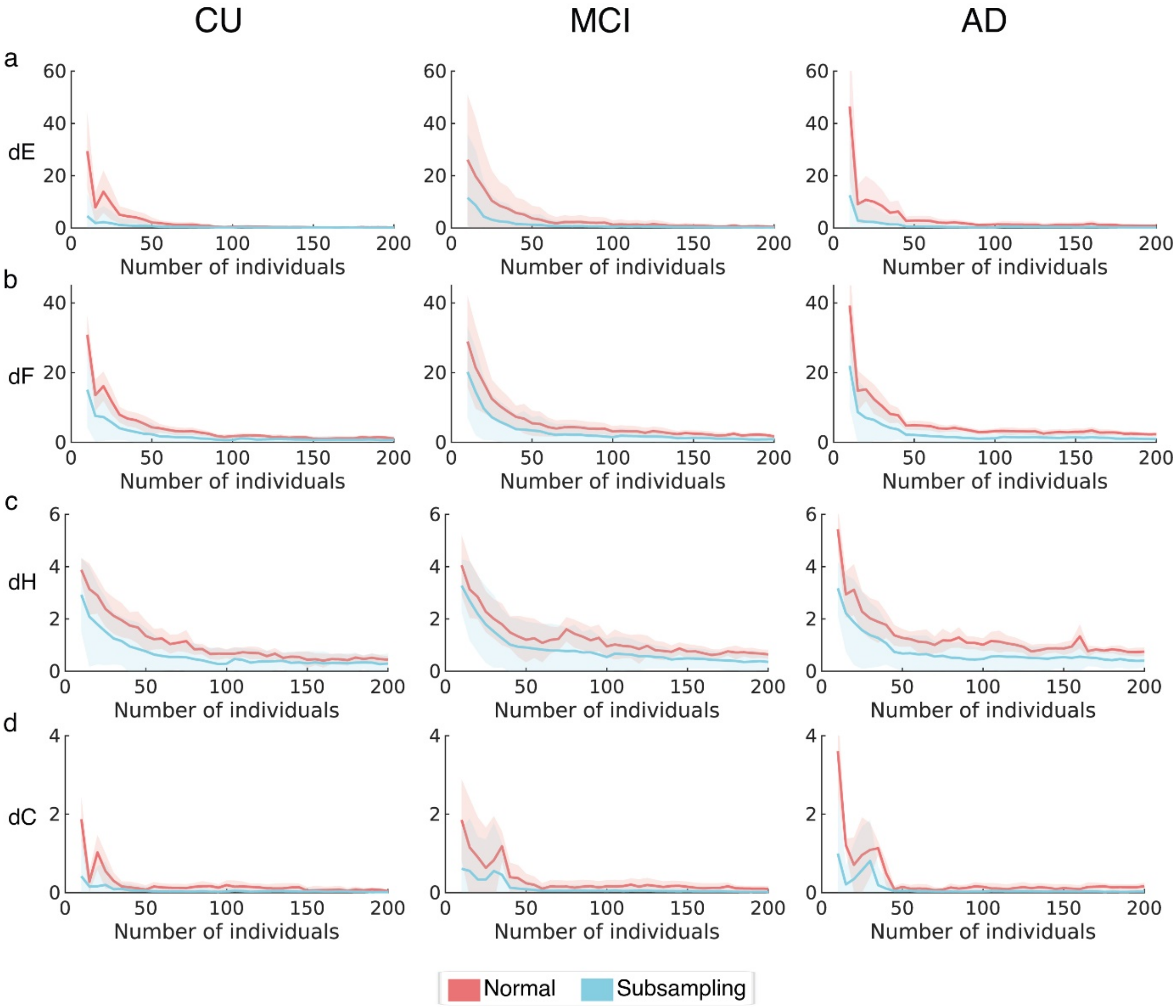

Supplemental Figure 10. Comparison between the conventional and the MS subsampling method as a function of the dataset size and 2% outlier attack. Groups CU, MCI, and AD were network outlier attacked 256 times (P_o = 2%) for each dataset size defined in the interval [10, 15, 20, …, 200]. MS bootstrap MBNs were constructed using the mean matrix criterion, α = 0.0001, and n was set to 9,300. The stability of MBNs was evaluated using dE, dF, dH, and dC, shown in panels (a), (b), (c), and (d) for both conventional and bootstrap methods. Bold lines represent the mean stability measure values, while the light shadows indicate three times the standard deviation from the mean.

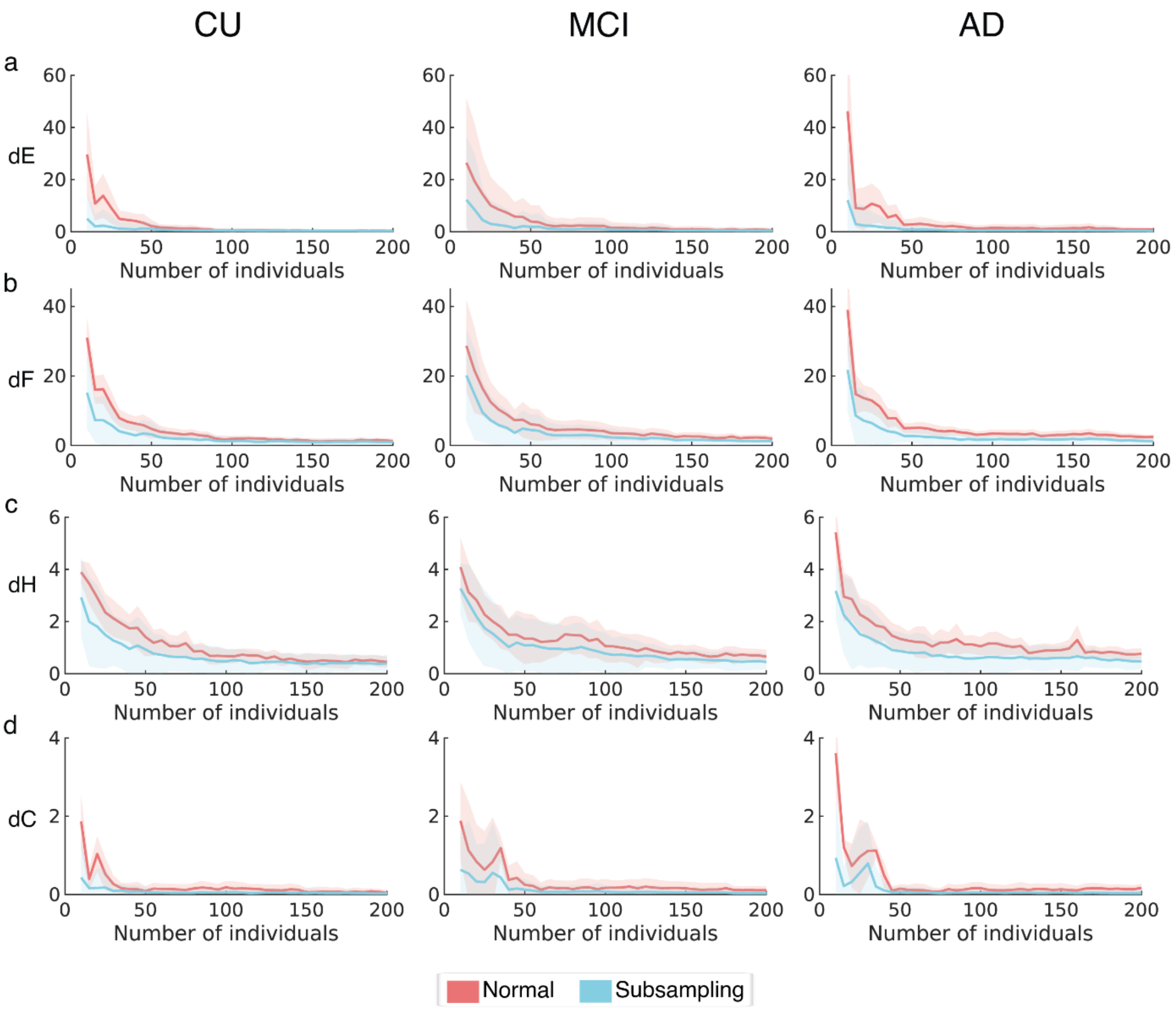

Supplemental Figure 11. Comparison between the conventional and the MS subsampling method as a function of the dataset size and 5% outlier attack. Groups CU, MCI, and AD were network outlier attacked 256 times (P_o = 5%) for each dataset size defined in the interval [10, 15, 20, …, 200]. MS bootstrap MBNs were constructed using the mean matrix criterion, α = 0.0001, and n was set to 9,300. The stability of MBNs was evaluated using dE, dF, dH, and dC, shown in panels (a), (b), (c), and (d) for both conventional and bootstrap methods. Bold lines represent the mean stability measure values, while the light shadows indicate three times the standard deviation from the mean.

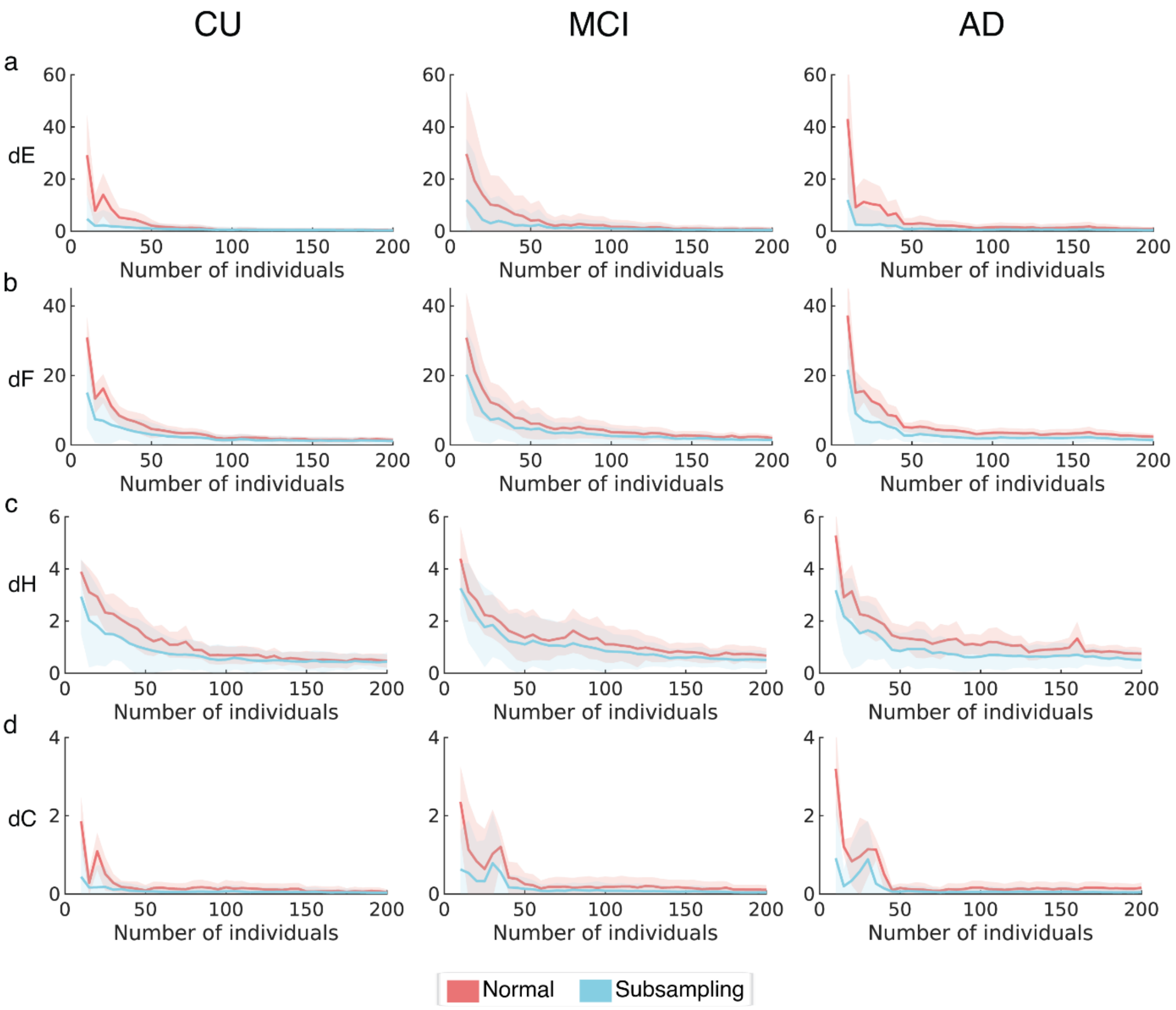

Supplemental Figure 12. Comparison between the conventional and the MS subsampling method as a function of the dataset size and 8% outlier attack. Groups CU, MCI, and AD were network outlier attacked 256 times (P_o = 8%) for each dataset size defined in the interval [10, 15, 20, …, 200]. MS bootstrap MBNs were constructed using the mean matrix criterion, α = 0.0001, and n was set to 9,300. The stability of MBNs was evaluated using dE, dF, dH, and dC, shown in panels (a), (b), (c), and (d) for both conventional and bootstrap methods. Bold lines represent the mean stability measure values, while the light shadows indicate three times the standard deviation from the mean.

Table S1. Volumes of interest.

Adopted abbreviation		Brain region
SFG_R	SFG_L	*Right and left "superior frontal gyrus"*
MFG_R	MFG_L	*Right and left "middle frontal gyrus"*
InfFG_R	InfFG_L	*Right and left "inferior frontal gyrus"*
MedFG_R	MedFG_L	*Right and left "medial frontal gyrus"*
MFOG_R	MFOG_L	*Right and left "medial front-orbital gyrus"*
LatFOG_R	LatFOG_L	*Right and left "lateral front-orbital gyrus"*
PreCG_R	PreCG_L	*Right and left "precentral gyrus"*
CR_R	CR_L	*Right and left "cingulate region"*
PostCG_R	PostCG_L	*Right and left "postcentral gyrus"*
SupPL_R	SupPL_L	*Right and left "superior parietal lobule"*
SupMg_R	SupMg_L	*Right and left "supramarginal gyrus"*
AngG_R	AngG_L	*Right and left "angular gyrus"*
Precuneus_R	Precuneus_L	*Right and left "precuneus"*
Ins_R	Ins_L	*Right and left "insula"*
SupTG_R	SupTG_L	*Right and left "superior temporal gyrus"*
MTG_R	MTG_L	*Right and left "middle temporal gyrus"*
InfTG_R	InfTG_L	*Right and left "inferior temporal gyrus"*
MedOTG_R	MedOTG_L	*Right and left "medial occipitotemporal gyrus/fusiform"*

LatOTG_R	LatOTG_L	*Right and left "lateral occipitotemporal gyrus/fusiform"*
ParahipG_R	ParahipG_L	*Right and left "parahippocampal gyrus"*
Uncus_R	Uncus_L	*Right and left "uncus"*
SupOG_R	SupOG_L	*Right and left "superior occipital gyrus"*
MOG_R	MOG_L	*Right and left "middle occipital gyrus"*
InfOG_R	InfOG_L	*Right and left "inferior occipital gyrus"*
Cuneus_R	Cuneus_L	*Right and left "cuneus"*
LingG_R	LingG_L	*Right and left "lingual gyrus"*
OP_R	OP_L	*Right and left "occipital pole"*
Cerebellum_R	Cerebellum_L	*Right and left "cerebellum"*
HipForm_R	HipForm_L	*Right and left "hippocampal formation"*
Amyg_R	Amyg_L	*Right and left "amygdala"*
Thal_R	Thal_L	*Right and left "thalamus"*
SubThal_R	SubThal_L	*Right and left "subthalamic nucleus"*
P_R	P_L	*Right and left "putamen"*
Gpal_R	Gpal_L	*Right and left "globus palladus"*
CN_R	CN_L	*Right and left "caudate nucleus"*
Nacc_R	Nacc_L	*Right and left "nucleus accumbens"*

Table S2. Two-way ANOVA results using ADNI cohort – MBN stability evaluation with different data imbalance setups

ADASYN				
**Effect**	**Stability Meaure**			
	**dE**	**dH**	**dF**	**dC**
**Group**	F (2, 1530) = 17223, p < 0.0001	F (2, 1530) = 329.0, p < 0.0001	F (2, 1530) = 3103, p < 0.0001	F (2, 1530) = 8043, p < 0.0001
**Method**	F (1, 1530) = 72152, p < 0.0001	F (1, 1530) = 31514, p < 0.0001	F (1, 1530) = 32067, p < 0.0001	F (1, 1530) = 126559, p < 0.0001
**Interaction**	F (2, 1530) = 14068, p < 0.0001	F (2, 1530) = 20.00, p < 0.0001	F (2, 1530) = 1541, p < 0.0001	F (2, 1530) = 11909, p < 0.0001

Undersampled				
**Effect**	**Stability Meaure**			
	**dE**	**dH**	**dF**	**dC**
**Group**	F (2, 1530) = 243.6, p < 0.0001	F (2, 1530) = 99.01, p < 0.0001	F (2, 1530) = 367.0, p < 0.0001	F (2, 1530) = 1009, p < 0.0001

**Method**	F (1, 1530) = 70006, p < 0.0001	F (1, 1530) = 65796, p < 0.0001	F (1, 1530) = 89860, p < 0.0001	F (1, 1530) = 59375, p < 0.0001
**Interaction**	F (2, 1530) = 117.2, p < 0.0001	F (2, 1530) = 447.6, p < 0.0001	F (2, 1530) = 11.96, p < 0.0001	F (2, 1530) = 744.1, p < 0.0001

**Imbalanced**				
**Effect**	**Stability Meaure**			
	**dE**	**dH**	**dF**	**dC**
**Group**	F (2, 1530) = 16141, p < 0.0001	F (2, 1530) = 949.7, p < 0.0001	F (2, 1530) = 3561, p < 0.0001	F (2, 1530) = 6811, p < 0.0001
**Method**	F (1, 1530) = 81220, p < 0.0001	F (1, 1530) = 69351, p < 0.0001	F (1, 1530) = 29194, p < 0.0001	F (1, 1530) = 48148, p < 0.0001
**Interaction**	F (2, 1530) = 12525, p < 0.0001	F (2, 1530) = 66.77, p < 0.0001	F (2, 1530) = 1550, p < 0.0001	F (2, 1530) = 4306, p < 0.0001

Group factor with 3 levels: CU, MCI, AD

Method factor with 2 levels: Conventional, Bootstrap
